# Synaptic Origins of the Complex Receptive Field Structure in Primate Smooth Monostratified Retinal Ganglion Cells

**DOI:** 10.1523/ENEURO.0280-23.2023

**Published:** 2024-01-25

**Authors:** Sara S. Patterson, Rebecca J. Girresch, Marcus A. Mazzaferri, Andrea S. Bordt, Wendy L. Piñon-Teal, Brett D. Jesse, Dinukie-Chantal W. Perera, Melanie A. Schlepphorst, James A. Kuchenbecker, Alice Z. Chuang, Jay Neitz, David W. Marshak, Judith Mosinger Ogilvie

**Affiliations:** ^1^Center for Visual Science, University of Rochester, Rochester, NewYork 14617; ^2^Department of Biology, Saint Louis University, Saint Louis, Missouri 63103; ^3^Department of Ophthalmology, University of Washington, Seattle, Washington 98104; ^4^Departments of Ophthalmology & Visual Science, McGovern Medical School, Houston, Texas 77030; ^5^Neurobiology and Anatomy, McGovern Medical School, Houston, Texas 77030

**Keywords:** connectomics, parallel processing, primate, retinal circuitry, retinal ganglion cell, vision

## Abstract

Considerable progress has been made in studying the receptive fields of the most common primate retinal ganglion cell (RGC) types, such as parasol RGCs. Much less is known about the rarer primate RGC types and the circuitry that gives rise to noncanonical receptive field structures. The goal of this study was to analyze synaptic inputs to smooth monostratified RGCs to determine the origins of their complex spatial receptive fields, which contain isolated regions of high sensitivity called “hotspots.” Interestingly, smooth monostratified RGCs co-stratify with the well-studied parasol RGCs and are thus constrained to receiving input from bipolar and amacrine cells with processes sharing the same layer, raising the question of how their functional differences originate. Through 3D reconstructions of circuitry and synapses onto ON smooth monostratified and ON parasol RGCs from central macaque retina, we identified four distinct sampling strategies employed by smooth and parasol RGCs to extract diverse response properties from co-stratifying bipolar and amacrine cells. The two RGC types differed in the proportion of amacrine cell input, relative contributions of co-stratifying bipolar cell types, amount of synaptic input per bipolar cell, and spatial distribution of bipolar cell synapses. Our results indicate that the smooth RGC's complex receptive field structure arises through spatial asymmetries in excitatory bipolar cell input which formed several discrete clusters comparable with physiologically measured hotspots. Taken together, our results demonstrate how the striking differences between ON parasol and ON smooth monostratified RGCs arise from distinct strategies for sampling a common set of synaptic inputs.

## Significance Statement

Vision begins in the retina where the photoreceptor outputs are processed and then conveyed to the brain by 20 or more ganglion cell types, each carrying a distinct message about the spatial, temporal, and spectral properties of incoming light. Understanding the contents of these messages and the retinal circuitry that generate them is a key goal for visual neuroscience. Here we used 3D reconstructions to map the upstream circuitry for the rare ON smooth monostratified ganglion cells and the more common, well-studied parasol ganglion cells. We find the two ganglion cell types employ different sampling strategies to extract distinct types of visual information from a common set of synaptic inputs.

## Introduction

Retinal ganglion cells (RGCs) provide the sole source of visual information to the primate brain. The primate retina contains at least 20 RGC types, each conveying different information about the spatiotemporal pattern of light intensity and wavelength on the retina (Dacey et al., 2003; Dacey, 2004; Field and Chichilnisky, 2007; Grünert and Martin, 2020). A long-standing question in visual neuroscience is how the photoreceptor outputs are processed in parallel to generate diverse responses at the level of the retinal output (Wässle, 2004; Gollisch and Meister, 2010). This parallel processing begins at the first synapse of vision, where photoreceptor outputs diverge onto at least 12 morphological types of bipolar cells, each differing in their temporal, spatial, and spectral properties (Euler et al., 2014; Tsukamoto and Omi, 2016). RGCs receive excitatory input from bipolar cells within the inner retina, along with predominantly inhibitory input from amacrine cells (Diamond, 2017; Yan et al., 2020). Circuitry within the inner retina is highly organized, with each cell type stratifying in specific layers that differ in their functional properties (Roska and Werblin, 2001; Euler et al., 2014). As a result, the stratification of each RGC's dendritic field constrains the available synaptic inputs and, thus, the visual information available for transmission to the brain.

The focus of this study is to understand how distinct response properties emerge in co-stratifying ON parasol and ON smooth monostratified RGCs that sample from a shared set of synaptic inputs. ON parasol RGCs are among the most common and best-studied primate RGC types. In contrast, little is known about the ON smooth monostratified RGC circuitry. Recently, striking differences in the spatial receptive fields of smooth monostratified and parasol RGCs have been described (Rhoades et al., 2019). The parasol RGCs have a relatively homogeneous center–surround receptive field, while the smooth monostratified RGCs have a complex receptive field with isolated regions of increased light sensitivity, or “hotspots,” separated by regions with lower sensitivity, referred to here as “coolspots.” Interestingly, the hotspots and coolspots within each smooth monostratified RGC's receptive field align with the receptive fields of simultaneously measured parasol RGCs, suggesting a relationship between the dendritic fields of the two RGC types. The origins of the unique receptive field structure of smooth monostratified RGC are unknown.

We hypothesized that the spatial receptive field structure of smooth monostratified RGC could be explained by spatial asymmetries in the bipolar cell input to their dendritic fields. To test this hypothesis, we reconstructed both ON smooth monostratified and ON parasol RGCs and then analyzed their synaptic inputs. First, we found that ON smooth monostratified RGCs receive a higher proportion of amacrine input than ON parasol RGCs. Second, we classified the presynaptic bipolar cell types for both ON smooth monostratified and ON parasol RGCs. The two RGC types received different proportions of input from DB4, DB5, and giant bipolar cells while only the parasol RGCs received midget bipolar cell input. The density of presynaptic bipolar cells and the number of synapses per bipolar cell were much higher for the parasol RGCs. Finally, the spatial distribution of bipolar cell input to the ON smooth monostratified RGCs formed distinct clusters consistent with the hotspots measured physiologically (Rhoades et al., 2019). In contrast, synapses onto ON parasol RGCs had a more homogeneous distribution. Taken together, these results suggest an explanation for the unusual receptive field structure of smooth monostratified RGCs.

## Materials and Methods

### Electron microscopy

Retinal tissue was obtained from a terminally anesthetized, adult male macaque (*Macaca nemestrina*) through the Tissue Distribution Program at the Washington National Primate Center. All procedures were approved by the Institutional Animal Care and Use Committee at the University of Washington. Central retinal tissue was processed for serial block-face scanning electron microcopy (SBF-SEM) as described previously (Patterson et al., 2019). Briefly, a 1 × 1 mm^2^ ∼1 to 1.5 mm inferior to the center of the fovea was fixed, stained en bloc with heavy metals, and embedded in epoxy resin. The images were acquired using a Zeiss Sigma VP field emission scanning electron microscope with a 3View system (Gatan).

Tissue preparation and SEM imaging were optimized in signal-to-noise ratio for visualization of small, low-contrast features such as synaptic ribbons (Patterson et al., 2022).The retinal tissue was sectioned in the horizontal plane at 90 nm thickness and imaged as a 5 × 5 montage, producing an area ∼200 μm on each side with a resolution of 7.5 nm/pixel. The resulting volume contained 1,893 sections, spanning from the ganglion cell layer (GCL) through the cone pedicles, and has been used in several previous studies (Patterson et al., 2019, 2020a,b, 2022; Bordt et al., 2022). Image registration was performed using Nornir (http://nornir.github.io; RRID: SCR_003584), and the image tiles were reassembled into cohesive digital volumes and hosted on a 24-core server at the University of Washington.

### Connectomic reconstruction

The serial EM volume was annotated using the web-based, multiuser Viking software described previously (Anderson et al., 2011; http://connectomes.utah.edu; RRID: SCR_005986). Briefly, profiles of processes were annotated by placing circular disks with the same diameter at their centers of mass. Synaptic densities were annotated with lines and linked to the neurons in which they were located. Examples of amacrine and bipolar cell synapses are shown in Extended Data Figures 4-1, 6-1, 10-1, and 10-2.

Bipolar cell synapses were identified by the presence of a synaptic ribbon and halo of vesicles, associated with postsynaptic membrane densities. Amacrine cell synapses were identified by a cluster of vesicles associated with membrane densities. In both cases, features were present in at least two consecutive sections. These criteria were established to minimize false positives, but they do allow for the possibility that very small synapses were omitted from the analysis.

**Figure 1.**
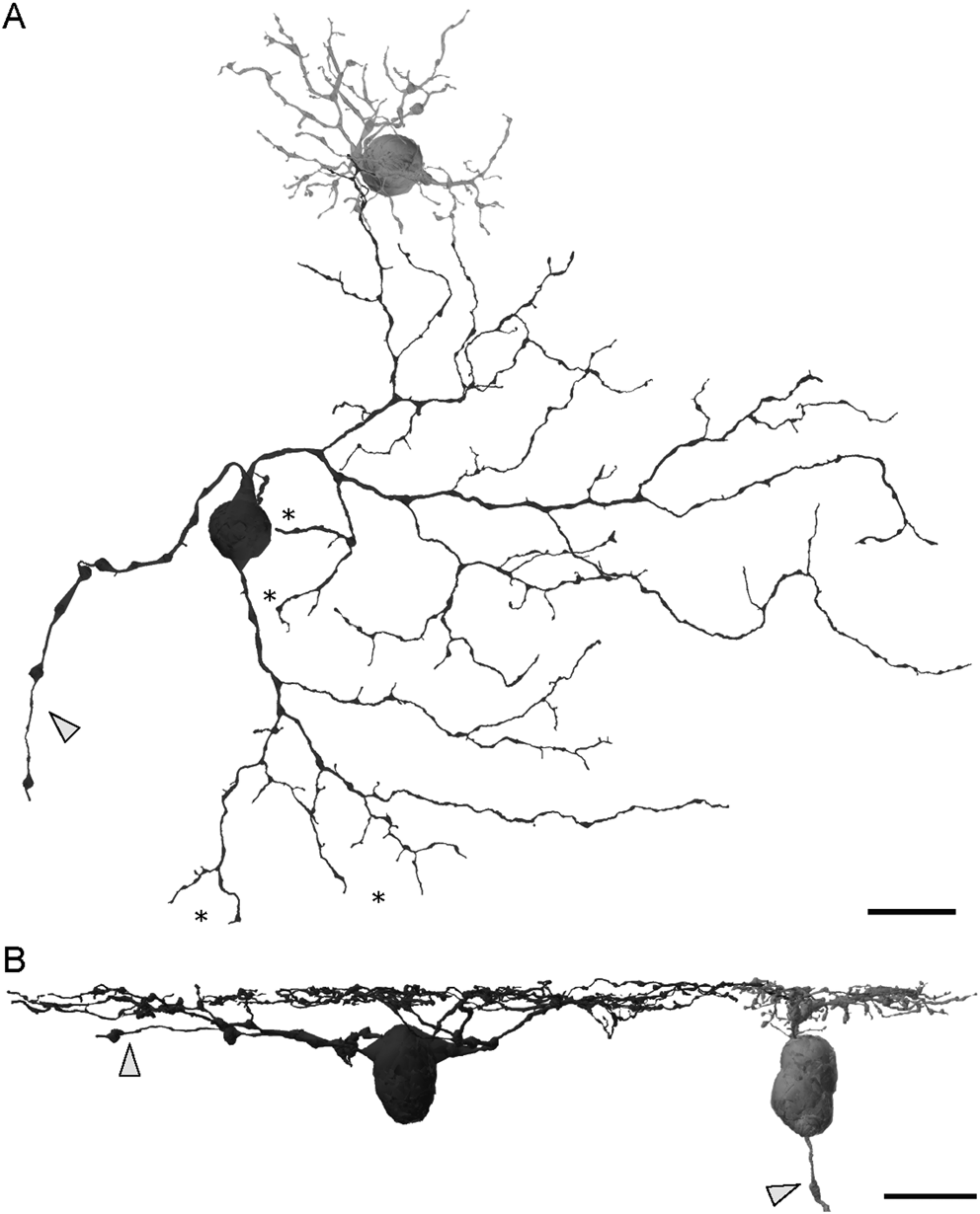
3D Reconstruction of an ON smooth monostratified RGC (dark gray) and an ON parasol RGC (light gray). ***A***, The somas are of comparable size, but the two cell types can be distinguished by the size, density, and branching pattern of their dendrites. ***B***, Side view of the same RGCs showing co-stratification of their dendrites. Arrows indicate axons and asterisks indicate areas where the smooth RGC’s dendrites reached the volume’s edge. Scale bar, 20 µm. Dendritic field diameters for the full population of parasol and smooth RGCs are included in Extended Data Table 1-1.

10.1523/ENEURO.0280-23.2023.f1-1 Extended Data Figure 4-1. Bipolar cell inputs to an ON smooth monostratified RGC (yellow). A. DB4 bipolar cell (pink), B. DB5 bipolar cell (blue), C. Giant bipolar cell (orange). Synaptic ribbons are indicated by black arrowheads. Scale bar = 1 μm. Download Figure 4-1, TIF file.

10.1523/ENEURO.0280-23.2023.f6-1 Extended Data Figure 6-1. Bipolar cell inputs to ON parasol RGC dendrites (dark yellow). A. DB4 bipolar cell (pink), B. DB5 bipolar cell (blue), C. DB6 bipolar cell (bright yellow), D. Giant bipolar cell (orange), D. ON midget bipolar cell (green). Synaptic ribbons are indicated by black arrowheads. Scale bar = 1 μm. Download Figure 6-1, TIF file.

10.1523/ENEURO.0280-23.2023.f10-1 Extended Data Figure 10-1. Example synapses from a candidate coolspot within the smooth monostratified RGC's dendritic field shown in serial sections. Arrows mark DB4 synapses onto two parasols (5063 and 18269) in the 2nd and 4th rows. The smooth RGC receives no bipolar cell input. A representative amacrine cell synapse onto the smooth RGC is marked with an arrow in the 3rd row. Download Figure 10-1, TIF file.

**Figure 2.**
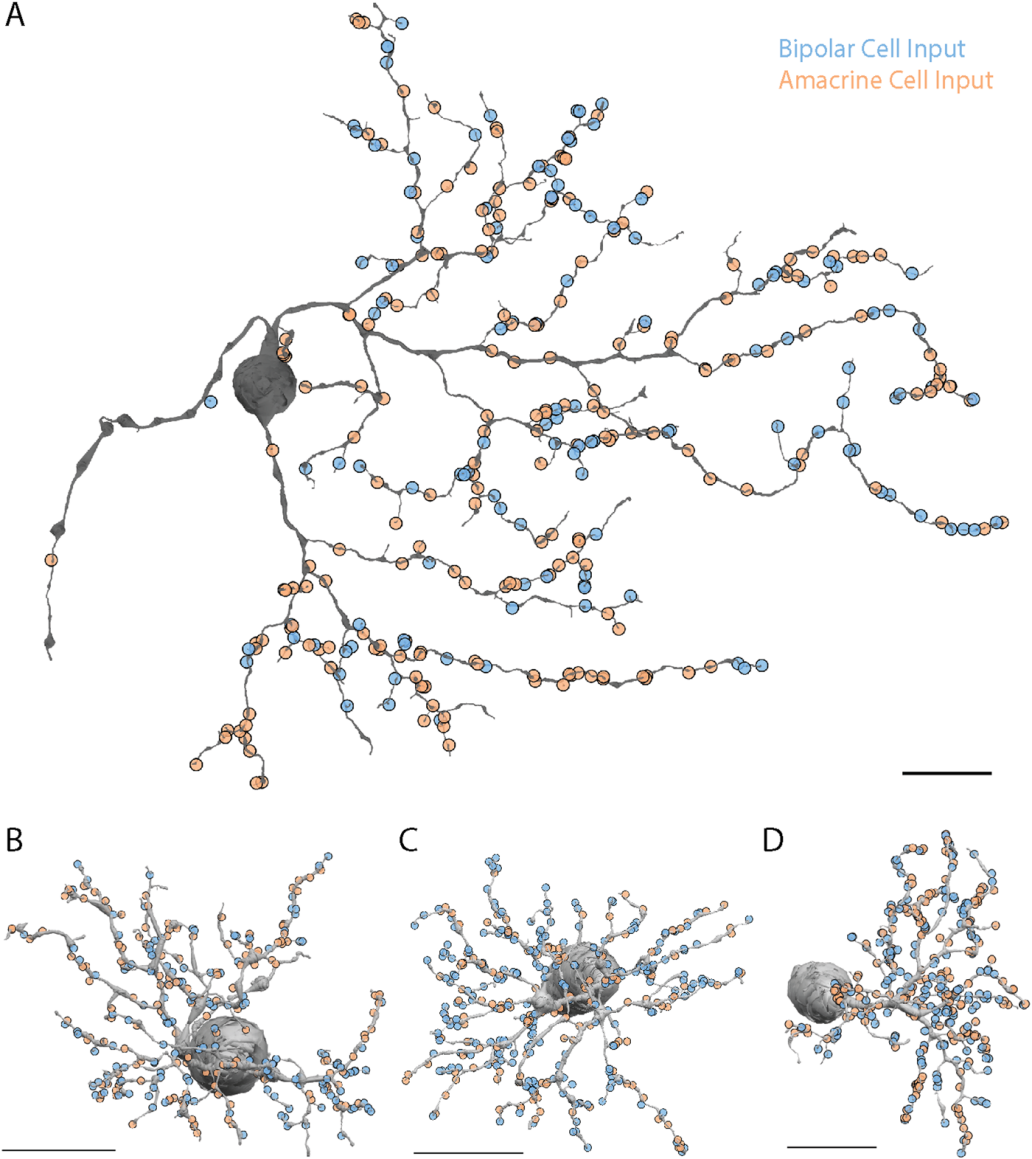
Synaptic inputs to an ON smooth monostratified RGC (***A***) and three parasol RGCs (IDs: 5370, 18269 and 5063, respectively). ***B–D***, Amacrine cell inputs are marked in orange and bipolar cell inputs in blue. Scale bar, 20 µm.

10.1523/ENEURO.0280-23.2023.f10-2 Extended Data Figure 10-2. An example synapse from a DB5 bipolar cell onto the smooth RGC within a hotspot region of the dendritic field shown in serial sections. A short off-shoot branch from the smooth RGC extends towards the bipolar cell and ends abruptly after receiving input from a ribbon synapse (arrow). The bottom row shows the location of the diffuse bipolar cell within the smooth RGC's dendritic field. Bottom left scale bar = 20 μm, bottom right scale bar = 5 μm. Download Figure 10-2, TIF file.

The major cell types (amacrine cells, bipolar cells, and ganglion cells) and synapses (conventional and ribbon) were identified using established ultrastructural criteria (Dowling and Boycott, 1966). Bipolar cell types were classified based on previously described anatomical features (Boycott and Wassle, 1991; Joo et al., 2011; Tsukamoto and Omi, 2016; Patterson et al., 2022). Specifically, ON midget bipolar cells were identified by their small, compact axonal arbors that terminated at 77% depth in the IPL, on average. Giant bipolar cells were identified by their large, sparse axonal arbor and low density of synaptic outputs. A DB6 bipolar cell was distinguished from DB4 and DB5 bipolar cells by its larger axonal arbor that stratified at 93.6% depth in the IPL. DB4 and DB5 bipolar cells both stratified at 58% depth but could be distinguished by their characteristic morphology and mosaic patterning. While DB4 bipolar cells had less varicose arbors than DB5 bipolar cells, DB5 terminals were more bulbous in appearance. Although these morphological differences were subtle in some reconstructed DB4 and DB5 bipolar cells, further confirmation of DB4 vs DB5 classification was obtained by analyzing the mosaics formed by each bipolar cell type. Bipolar cell terminals of the same type form regular arrays, while those of different types overlap (Tsukamoto and Omi, 2016).

### Data analysis and visualization

Data analysis and 3D rendering were performed in MATLAB (MathWorks; RRID: SCR_001622), on a Windows PC using SBFSEM-tools (https://github.com/neitzlab/sbfsem-tools; RRID: SCR_017350), an open-source connectomics toolbox. RGC soma diameters were analyzed using the SomaDiameter function; stratification depth of RGC dendrites and bipolar cell axon terminals were analyzed using the IPLDepth function (Patterson et al., 2020a). The boundary between the inner nuclear layer (INL) and the inner plexiform layer (IPL) was designated as 0% and the IPL-GCL boundary as 100% depth. Mean and mode IPL depth values were determined to be most accurate for RGC dendrites and bipolar cell axon terminals, respectively, as the long axon for each ON bipolar cell had a disproportionate impact on mean IPL depth calculations.

To determine the dendritic field diameter (Extended Data Table 1-1), we calculated the convex hull surrounding each dendritic field, that is, the 2D polygon bounding all annotations associated with each RGC (excluding the axon). Because some dendritic fields can be asymmetric, the reported diameters were calculated from the convex hull area.

Three additional statistical analyses were performed. A two-sample *t* test was used to compare the soma diameter and mean stratification depths of the two types of RGCs. A Fisher's exact test was used to compare the proportions of input from bipolar cells onto the two types of RGCs. The probability of *N* consecutive amacrine cell synapses onto an ON smooth monostratified RGC, uninterrupted by a bipolar cell synapse, was calculated using a geometric distribution, *p^N^*, where the probability of each amacrine cell synapse was determined by the ratio of total amacrine cell synapses to total synaptic input.

### Quantification and visualization of bipolar cell clusters

**Figure 3.**
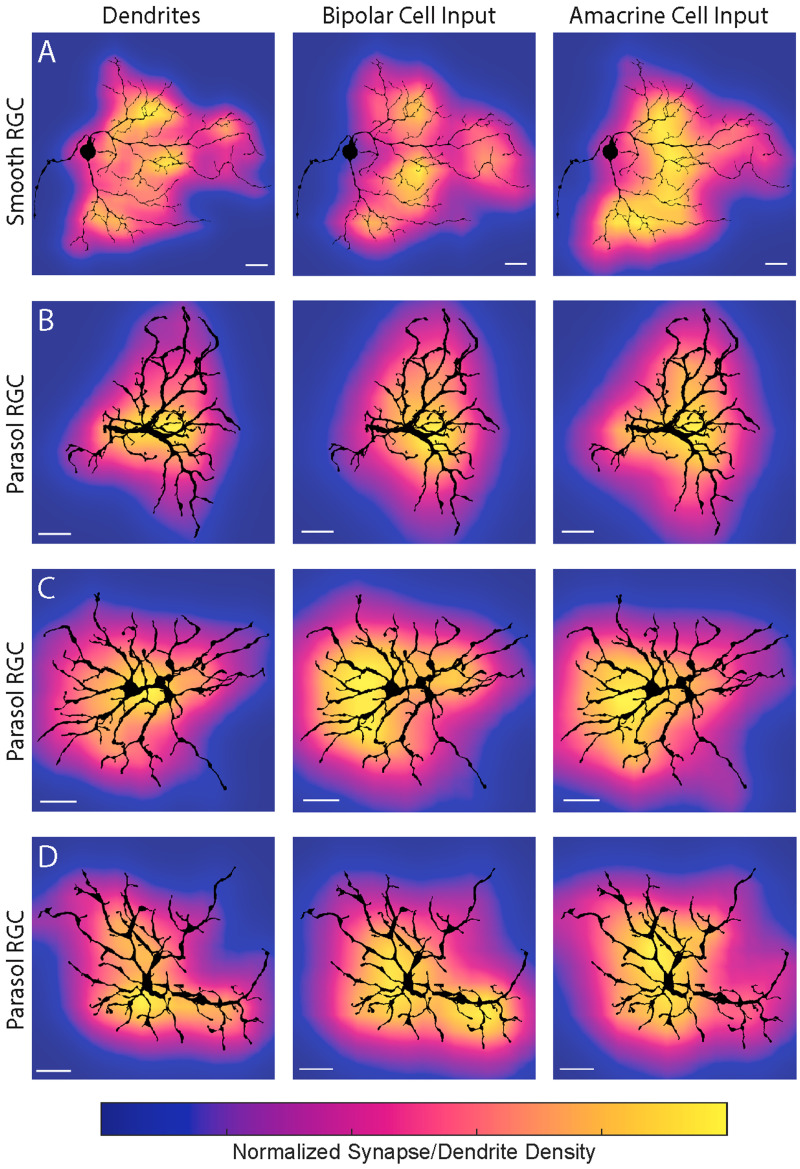
Density maps for smooth and parasol RGC dendrites, bipolar cell synapses and amacrine cell synapses. ***A***, The ON smooth monostratified RGC shows defined clusters of bipolar cell synapses separated by areas with minimal bipolar cell input. Scale bar, 10 µm. ***B–D***, The parasol RGCs’ bipolar cell input was more homogeneous and largely followed the dendrite density. The somas and axons of the parasol RGCs were removed to avoid obscuring the underlying density map. Scale bar, 20 µm. Each density map was obtained from smoothed 2D histograms (see Materials and Methods) and the color maps for each are independently normalized.

The density maps in Figures 3 and 8*A–C* were smoothed 2D histograms of the underlying annotations' *x*–*y* coordinates using the minimum smoothing value of *λ* = 1 (Eilers and Goeman, 2004). For the dendrite density plots, annotations associated with the soma and axon were omitted. A range of bin sizes were tested to determine a size that was small enough to reveal any potential underlying clusters but large enough to avoid producing discrete peaks for individual synapses or outlines of the dendrites. The bin size for the smooth RGC's histograms in Figures 3*A* and 8*A–C* was set to 9.7 μm for synapses and 6.3 μm for dendrites. For the parasol RGCs in Figure 3*B–D*, bin sizes varied slightly with dendritic field size, ranging from 4.5 to 4.7 μm for synapses and 3.3–3.5 μm for dendrites. The smaller bin size for the parasol RGCs was chosen to ensure any potential spatial clustering of bipolar cell input was not overlooked.

To quantify the hotspots within the smooth RGC's dendritic field and determine which synapses contributed to each hotspot, we clustered the *x* and *y* coordinates of the ribbon synapses using a mixture of Gaussians model. We tested models ranging from two to ten components and found that four components provided the best clustering of the data, as determined by the Calinski–Harabasz (CH) index (Caliñski and Harabasz, 1974). The CH index measures the similarity of a data point to its own cluster compared with the other clusters. For *K* clusters on the dataset $D=[d_{1}\;\;d_{2}\;\;\ldots \;\;d_{N}]>$, the CH index is defined as follows:

$$\mathrm{CH}=\;\left[\frac{\sum_{k=1}^{K}\;n_{k}\;{\left|\left|c_{k}-c\right|\right|}^{2}}{K-1}\right]/\;\left[\frac{\sum_{k=1}^{K}\;\sum_{k=1}^{n_{k}}\;{\left|\left|d_{i}-c_{k}\right|\right|}^{2}}{N-K}\right],$$

where *N* is the total number of data points, *c* is the global centroid, and *n_k_* and *c_k_* are the number of points and centroid of the *k*th cluster, respectively.

The model was used to cluster the bipolar cell synapses as shown in Figure 8*D*. The Mahalanobis distance metric to identify outliers is as follows:

$$d=\;\sqrt{(x-\mu )\sum^{-1}\;{\left(x-\mu \right)}^{\prime }},$$

where *μ* is the mean, ∑ is the covariance, and *d* is the distance of the vector *x* from the mean in standard deviations. Points greater than 3 SDs from the mean were excluded from the analysis in Figure 8*E*. The mean, standard deviation, and weight of each component is provided in Extended Data Table 8-1.

To classify the distance of the smooth RGC's bipolar cell inputs to the center of the parasol RGC's dendritic field in Figure 10*D*, we first obtained a convex hull for the parasol RGC's dendritic field as described above. Bipolar cell synapses to the smooth RGC whose *x*–*y* positions were located within the convex hull were classified as falling within the parasol RGC's dendritic field. The centroid of the convex hull was taken as the dendritic field center, and the distance of each synapse from this point was calculated as the 2D Euclidean distance.

### Figures

The figures were prepared using Adobe Photoshop and Adobe Illustrator. The color palette was selected so that the figures could be interpreted by individuals with all of the common forms of color blindness (http://mkweb.bcgsc.ca/colorblind/). SBFSEM-tools was used to produce 3D renders of annotated neurons and scatterplots of synapse locations as described in our previous work (Bordt et al., 2019).

The underlying EM data and annotations are freely available for viewing with Viking using http://v2486.host.s.uw.edu/Neitz/InferiorMonkey/SliceToVolume.vikingxml. Each cell can be accessed through their unique ID number. The primary ON smooth monostratified RGC used throughout the study and figures is 1321. The second ON smooth monostratified RGC included in analyses as indicated in the text is 7889. Five parasol RGCs were reconstructed: 5035, 5063, 5370, 18269, and 21392. Synaptic inputs to three of these parasol RGCs (5370, 18269, and 5063) were analyzed in detail and are shown in Figure 2*B*,*C*,*D*, respectively. Combined data from all three RGCs is included in Figures 5*B* and 7*B*. Parasol RGC 18269 is included in Figures 2*C*, 3*C*, 6, 9, and 10, Extended Data Tables 5-1, 5-2, 7-3, and Extended Data Figures 6-1*C* and 10-1. Parasol RGC 5370 is included in Figures 1, 2*B*, and 3*D*, Extended Data Figures 6-1*A*, 6-1*B*, 6-1*D*, 6-1*E*, and Extended Data Tables 5-1, 5-2, and 7-2. Parasol RGC 5063 is included in Figures 2*D*, 3*B*, and 9, Extended Data Tables 5-1, 5-2, 7-4, and Extended Data Figure 10-1. The three parasol RGCs located within the smooth RGC's dendritic field in Figure 9 are 18269, 5063, and 5035. The specific bipolar cells shown in Extended Figure 4-1*A–C* are 51073, 22499, and 27097, respectively. The bipolar cells shown in Extended Figure 6-1*A–E* are 9198, 12767, 11043, 21768, and 9612, respectively. The DB4 bipolar cell in Extended Data Figure 10-2 is 51063, and the DB4 bipolar cell shown in Figure 10*C* and Extended Data Figure 10-1 is 19331. Other bipolar cells can be located by visually scanning through the cells in the EM volume in Viking for cells labeled by each bipolar cell type or querying the cell labels using SBFSEM-tools.

10.1523/ENEURO.0280-23.2023.t1-1 Extended Data Table 1-1. Dendritic field diameters for parasol and smooth monostratified RGCs. Download Table 1-1, DOCX file.

10.1523/ENEURO.0280-23.2023.t5-1 Extended Data Table 5-1. Total presynaptic bipolar cells for smooth monostratified RGC (1321) and three parasol RGCs (5063, 5370, 18269) by type. IMB stands for invaginating ("ON") midget bipolar cell. Download Table 5-1, DOCX file.

10.1523/ENEURO.0280-23.2023.t5-2 Extended Data Table 5-2. Total bipolar cell synapses by type for smooth monostratified RGC (1321) and three parasol RGCs (5063, 5370, 18269) by type. Download Table 5-2, DOCX file.

### Code accessibility

The code/software described in the paper is freely available online at https://github.com/sarastokes/SmoothMonostratifiedRGC.git. The code is available as Extended Data.

10.1523/ENEURO.0280-23.2023.d1 Extended Data 1 Code used to create figures and analyze the underlying annotation data. Download Data 1, ZIP file.

## Results

### Morphology of ON smooth monostratified and ON parasol RGCs

We reconstructed two ON smooth monostratified RGCs and five ON parasol RGCs, which will be referred to as smooth and parasol RGCs for simplicity. We focused our analysis of synaptic inputs on the most completely reconstructed smooth RGC and three parasol RGCs. The smooth and parasol RGCs displayed clear morphological differences, as expected from previous work (Yamada et al., 2005; Crook et al., 2008a). Smooth RGCs had proximal dendrites that branched in a radiate pattern and distal dendrites that were relatively straight and lacked spines. The dendrites of the parasol RGCs were more tortuous and had more spines.

The smooth RGC dendritic fields were considerably larger and less dense than those of the parasol RGCs. The dendritic field diameters of the smooth RGCs were >2.7× larger than those of the parasol RGCs (153.1 ± 12.6 μm vs 57.0 ± 2.1 μm; *n* = 2, 5; Fig. 1 and Extended Data Table 1-1). Importantly, the smooth RGC diameters are likely underestimates as a portion of their dendritic fields extended beyond the edge of the volume. In the peripheral retina, smooth RGCs are reported to have smaller somas than parasol RGCs (Crook et al., 2008a). However, in our sample from the central retina, the difference between smooth and parasol RGCs was not significant (14.5 ± 1.99 μm vs 14.9 ± 0.94 μm; *n* = 2, 5; *p* = 0.83).

We confirmed that the dendrites of the two RGC types co-stratify within the inner retina. The mean depth of their stratification was not significantly different (63.2 ± 0.71%, *n* = 2 for smooth RGCs and 64.6 ± 1.12%, *n* = 5, for the parasol RGCs; *p* = 0.28), confirming that both RGC types are spatially limited to receiving synaptic input from a common set of amacrine and bipolar cell types.

### Proportions and spatial distribution of synaptic input from amacrine and bipolar cells

We hypothesized that the smooth RGC's heterogeneous spatial receptive field arises through local asymmetries in excitatory and inhibitory synaptic input. The hotspots could result from regions with more excitatory bipolar cell input or less inhibitory amacrine cell input. Conversely, the coolspots could be created by regions with greater inhibitory input or less excitatory input. To distinguish between these potential explanations for the smooth RGC's receptive field structure, we mapped all amacrine and bipolar cell synapses onto the smooth RGCs (Fig. 2*A*). For comparison, we also mapped the synapses onto three parasol RGCs (Fig. 2*B–D*).

Amacrine cells provided the majority of the input to smooth RGCs. The proportion of amacrine input was significantly different between smooth and parasol RGCs (69.2 ± 10.7% vs 46.4 ± 5.9%; *n* = 2, 3; *p* < 0.0001), although this analysis is limited because the smooth RGC dendritic fields extended beyond the boundary of the volume. Bipolar cells provided the remainder of the synaptic input to both RGC types. Interestingly, the smooth RGC in Figure 2*A* received 5% fewer synapses than each of the parasol RGCs in Figure 2*A–C* despite having a much larger dendritic field size.

The bipolar cell input to the smooth RGC in Figure 2*A* appeared to be clustered and large gaps in bipolar cell input were apparent. To better visualize the spatial distribution of the bipolar and amacrine cell synapses, we mapped the synapses in Figure 2 as smoothed 2D histograms (Fig. 3). The bipolar cell input to the smooth RGC in Figure 3*A* formed four clusters resembling the hotspots measured in multielectrode array recordings (Rhoades et al., 2019).

How do the gaps in bipolar cell input to the smooth RGC arise? The morphology of retinal neurons is often tightly linked with its function and response properties (Kim et al., 2008; Grimes et al., 2010; Vlasits et al., 2016). Two features of the smooth RGC's morphology seen in Figure 1 are potentially relevant to the origin of hotspots and coolspots. First, the smooth RGC's primary dendrites branched directly around rather than above the soma. The resulting lack of dendritic branching immediately above the soma creates a gap in synaptic input to the dendritic field that could contribute to the coolspots measured physiologically (Brown et al., 2000; Rhoades et al., 2019). This structure contrasts with parasol RGCs where a primary dendrite extends from the soma prior to branching. Second, the smooth RGC's dendrites were generally sparse; however, smaller, thinner branches were observed in some, but not all, areas of the dendritic field. If the dendrite density were lower in the coolspots, there would be minimal opportunities for the smooth RGC to receive bipolar cell input. We found some correspondence between the dendrite density and clusters of bipolar cell synapses (Fig. 3*A*); however, dendrite density alone could not fully account for the locations and sizes of the bipolar cell synapse clusters. Taken together, these results are consistent with the hypothesis that hotspots correspond to regions of increased excitatory input from bipolar cells with dendrite density playing a secondary contributing role.

Amacrine cell input to the smooth RGC was homogeneous (Fig. 3*A*) but dominated in the regions between bipolar cell clusters. As such, inhibitory amacrine cell input could play a supporting role in establishing the coolspots within the smooth RGC's receptive field. Five or more consecutive synapses from amacrine cells were observed in 10 regions of the dendritic field of the smooth RGC (Fig. 2*A*). On one dendrite, amacrine cells provided 14 consecutive synapses, and on another dendrite with two terminal branches, amacrine cells provided the sole input with 13 synapses. This result would not be expected due to chance and indicates regions where bipolar cell inputs are largely absent. Given the 1.6:1 ratio of presynaptic amacrine to bipolar cells, the probability of five consecutive amacrine cell synapses is 8.8% and the probability of 14 is 0.1%.

The contiguous amacrine cell synapses, the majority of which are likely to be inhibitory, and the corresponding absence of excitatory bipolar cell input are expected to produce areas that are relatively insensitive to increments in light intensity, consistent with the coolspots in the smooth RGC's receptive field. Analogously, the increased excitatory input to the regions where bipolar cell synapses cluster and dendrite density is higher are predicted to result in spatially localized areas of greater light sensitivity, consistent with the hotspots in the smooth RGC's receptive field. In contrast, bipolar and amacrine cell input to the parasol RGCs was largely homogeneous (Fig. 3*B*–*D*) and predictable from the dendritic branching density.

### Bipolar cell input to ON smooth and ON parasol RGCs

**Figure 4.**
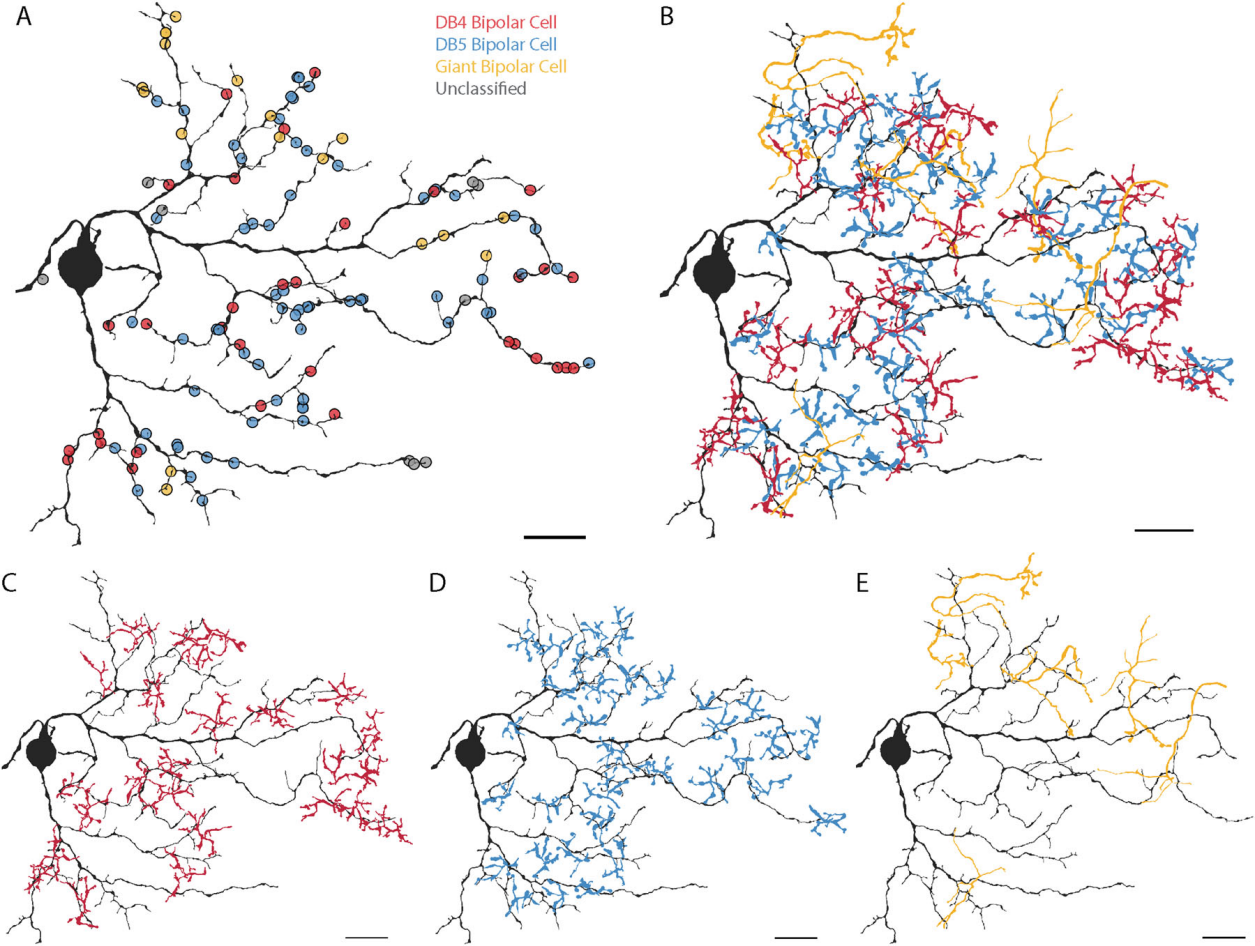
Bipolar cell input to a smooth monostratified RGC. ***A***, Locations of bipolar cell synapses colored by bipolar cell type. ***B***, The axon terminals of all classified bipolar cells presynaptic to the smooth RGC. ***C–E***, The smooth monostratified RGC and axon terminals of presynaptic DB4, DB5, and giant bipolar cells. Scale bar, 20 µm. Extended Data Figure 4-1 shows representative synapses from each bipolar cell type to the smooth RGC.

**Figure 5.**
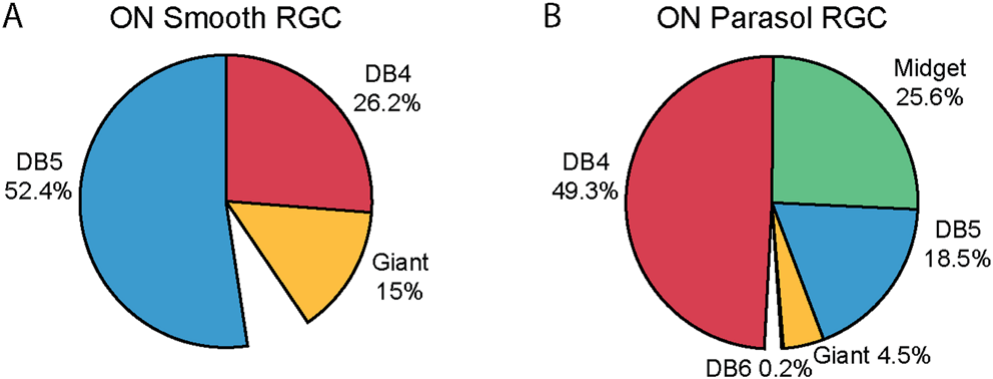
Relative proportion of DB4, DB5, DB6, giant bipolar, and ON midget bipolar cell input to (***A***) an ON smooth monostratified RGC (*n* = 126 synapses) and (***B***) three parasol RGCs (*n* = 559 synapses). The missing wedges in each pie chart represent synapses which could not be reliably classified (7.1% in ***A*** and 2.1% in ***B***). Extended Data Tables 5-1 and 5-2 provide numerical data for each RGC.

Because parasol and smooth monostratified RGCs co-stratify within the inner retina, they are limited to receiving input from the same set of co-stratifying bipolar cells: the DB4, DB5, ON midget, and giant bipolar cells. Parasol RGCs have been reported to receive input from all four types (Tsukamoto and Omi, 2016); however, the bipolar cell types providing input to smooth RGCs are unknown, so we next classified all presynaptic bipolar cells to the smooth RGC in Figure 2*A*. We were able to classify the presynaptic bipolar cells for 92.86% (117/126) of ribbon synapses onto the smooth RGC (Figs. 4*A*, 5*A*; see Materials and Methods). We aimed to address two questions with this classification. First, do smooth RGCs contact a different subset of bipolar cells than the parasol RGCs and/or have different proportions of inputs from the same bipolar cells? Physiological studies suggest each hotspot is made up of input from several bipolar types; however, the giant bipolar cell's axon terminals are similarly sized to the smooth RGC's hotspots. Second, which bipolar cell types contribute to the smooth RGC's hotspots?

We identified three different bipolar cell types presynaptic to the smooth RGC: DB4, DB5, and giant bipolar cells (Fig. 4). The majority of the ribbon synapse inputs were provided by DB5 bipolar cells with half as many synapses from DB4 bipolar cells and the remaining 15% from giant bipolar cells (Fig. 5*A*, Extended Data Tables 5-1, and 5-2). Representative electron micrographs of synapses from the three types of bipolar cells onto a smooth RGC are shown in Extended Data Figure 4-1. As expected from the bipolar synapse density map in Figure 3*A*, bipolar cell terminals were absent in several regions of the smooth RGC's dendritic field (Fig. 4*B*).

**Figure 6.**
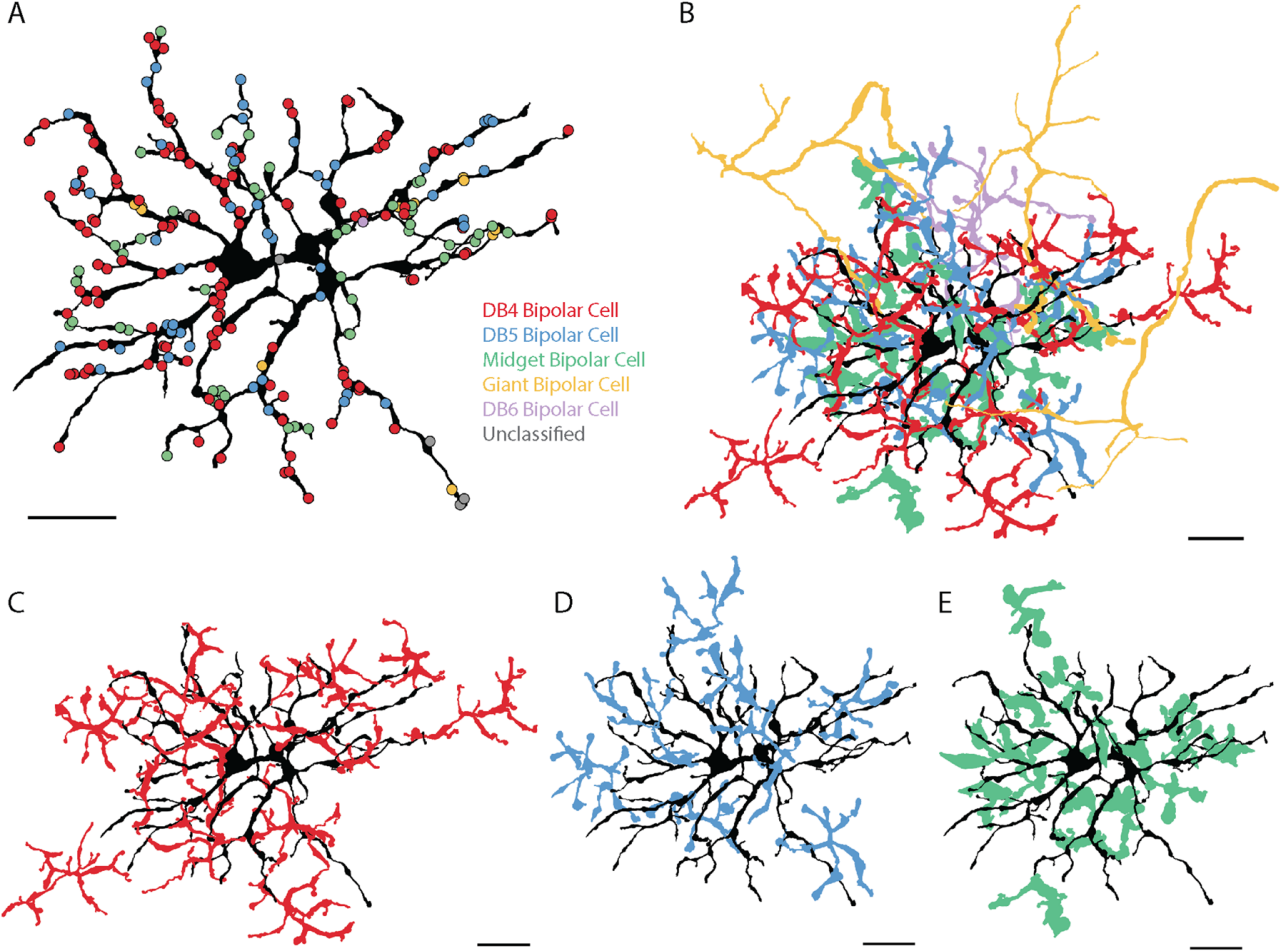
Bipolar cell input to an ON parasol RGC. ***A***, Locations of bipolar cell synapses colored by bipolar cell type. ***B***, The axon terminals of all classified presynaptic bipolar cells for the parasol RGC. ***C–E***, The parasol RGC and the axon terminals of the three most common presynaptic bipolar cell types: DB4, DB5, and ON midget bipolar cells, respectively. Scale bar, 20 µm. Extended Data Figure 6-1 shows representative synapses from each bipolar cell type onto parasol RGCs.

For comparison, we reconstructed the bipolar cell types presynaptic to three ON parasol RGCs in Figures 2*B–D*. Of the 559 bipolar cell synapses onto the three parasol RGCs, 547 (97.85%) could be reliably classified as a DB4, DB5, DB6, ON midget, or giant bipolar cell (Figs. 5*B*, 6*A*,*B*). DB4 bipolar cells provided approximately half of the parasol RGCs' bipolar cell input, ON midget bipolar cells provided 25.6%, DB4 bipolar cells provided 18.4%, and giant bipolar cells provided 5% (Fig. 5*B*, Extended Data Tables 5-1, and 5-2). In addition, a single synapse from a DB6 bipolar cell was observed onto one of the three parasol RGCs. Given the rarity (1/559 synapses) in our dataset and the absence of DB6 bipolar cell input to parasol RGCs in prior studies, this synapse is likely an outlier rather than a general feature of parasol RGCs. Representative electron micrographs of synapses from the five types of bipolar cells onto parasol RGCs are shown in Extended Data Figure 6-1. Unlike the smooth RGC, the parasol RGCs sampled from relatively complete mosaics of the three most common presynaptic bipolar cell types (Figs. 6*C*–*E*). These complete mosaics also increase confidence in our classification of DB4 and DB5 bipolar cells. Our classification of the parasol RGC's presynaptic bipolar cells was consistent with a previous report to mid-peripheral parasol RGCs (Tsukamoto and Omi, 2016); however, replicating this analysis in our more central volume enabled direct comparison with the smooth RGCs.

Each bipolar cell type conveys a different message from the photoreceptor array varying in its spatial, temporal, and spectral properties. In this way, bipolar cells have been called “building blocks” for downstream retinal computations (Euler et al., 2014), and the ways in which each RGC type combines their outputs plays a fundamental role in shaping their response properties. In this section, we identified several differences in how the smooth and parasol RGCs sample the outputs of co-stratifying bipolar cells. Both RGC types received input from DB4, DB5, and giant bipolar cells, but in different proportions. DB5s provided the dominant input to ON smooth monostratified RGCs, while DB4s provided the majority to ON parasol RGCs. Giant bipolar cells provided more input to the smooth RGC than the parasol RGCs. ON midget bipolar cells were the only co-stratifying bipolar cell type that did not contact both RGC types, providing substantial input to only the parasol RGCs.

### Sampling density of presynaptic bipolar cells

The most striking difference between parasol and smooth RGCs revealed by our bipolar cell reconstructions can be appreciated by comparing Figures 4*B* and 6*B*. The density of presynaptic bipolar cells to the parasol RGCs was much higher than that to the smooth RGC. While gaps in the mosaics of presynaptic bipolar cells for the smooth RGC were observed in several regions, the parasol RGCs received input from relatively complete mosaics of DB4, DB5, and ON midget bipolar cells. In contrast, there was minimal overlap and several gaps in the axon terminals of the smooth RGC's presynaptic bipolar cells.

Compared with the three parasol RGCs, the smooth RGC received fewer bipolar synapses (126 vs 186.3 ± 39.5; Extended Data Table 5-1) but had more presynaptic bipolar cells (69 vs 40.7 ± 3.1; Extended Data Table 5-2). If the number of synapses were evenly distributed among all presynaptic bipolar cell types, the smooth RGC would receive only 1.8 synapses per bipolar cell while the parasol would receive 4.57 synapses. How do the smooth and parasol RGCs distribute synapses across the different presynaptic bipolar cell types? To investigate, we quantified the number of synapses per presynaptic bipolar cell.

**Figure 7.**
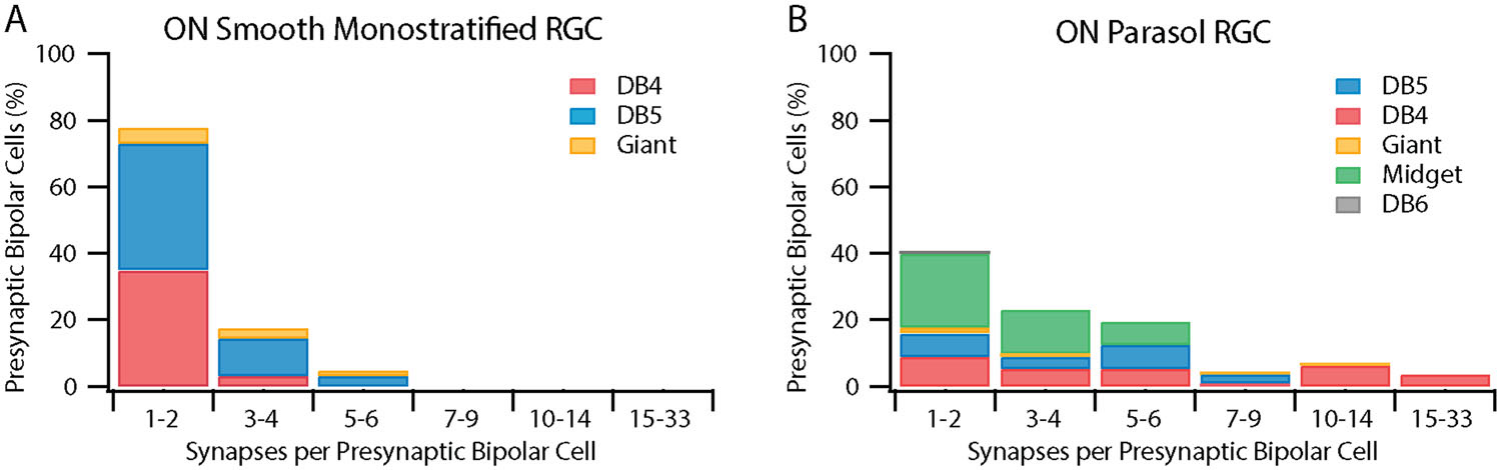
The number of synapses per presynaptic bipolar cell, sorted by bipolar cell type. ***A***, The smooth monostratified RGC in Figure 2*A* (*n* = 69 bipolar cells). ***B***, Three ON parasol RGCs in Figure 2*B–D* (*n* = 124 bipolar cells). The majority of the presynaptic bipolar cells for the smooth monostratified RGC made 1–4 synapses. In contrast, the parasol RGCs densely sampled from presynaptic bipolar cells. Extended Data Tables 7-1, 7-2, 7-3, and 7-4 provide numerical data for the smooth RGC and each parasol RGC.

We compared the amount of input from individual classified bipolar cells onto the smooth and parasol RGCs. Of the 63 identified bipolar cells presynaptic to the smooth RGC, 78% made just one or two synapses (Fig. 7*A*, Extended Data Table 7-1). No more than seven synapses from any single bipolar cell were observed and the cells providing three or more synapses were predominantly DB5 bipolar cells. This is in sharp contrast to bipolar cell input to parasol RGCs (Fig. 7*B*). Consistent with their smaller dendritic fields and larger total synaptic input, parasol RGCs received far more input from each presynaptic bipolar cell, with as many as 33 ribbon synapses from a single bipolar cell (Extended Data Tables 7-2, 7-3, and 7-4). Across the three parasol RGCs, 41% of bipolar cells presynaptic to parasol RGCs made just one or two synapses. Of those bipolar cells, over half were ON midget bipolar cells and many others were located along the distal-most dendrites of the parasol RGCs.

10.1523/ENEURO.0280-23.2023.t7-1 Extended Data Table 7-1. Number of ribbon synapses from each bipolar cell onto smooth RGC 1321. Download Table 7-1, DOCX file.

10.1523/ENEURO.0280-23.2023.t7-2 Extended Data Table 7-2. Number of ribbon synapses from each bipolar cell onto parasol RGC 5370. Download Table 7-2, DOCX file.

10.1523/ENEURO.0280-23.2023.t7-3 Extended Data Table 7-3. Number of ribbon synapses from each bipolar cell onto parasol RGC 18269. Download Table 7-3, DOCX file.

10.1523/ENEURO.0280-23.2023.t7-4 Extended Data Table 7-4. Number of ribbon synapses from each bipolar cell onto parasol RGC 5063. Download Table 7-4, DOCX file.

To summarize our results thus far, while the smooth and parasol RGCs' co-stratification constrains their dendrites to receiving input from the same presynaptic neurons, these data reveal four differences in their synaptic input that are predicted to distinguish the two RGC types functionally. First, smooth RGCs received more synaptic input from amacrine cells than bipolar cells while the opposite was observed for parasol RGCs. Second, bipolar cell synapses were clustered in several regions of the smooth RGC's dendritic field while the parasol RGCs' bipolar cell synapses were more homogeneous. Third, the two RGC types received different proportions of input from each bipolar cell type. Fourth, compared with the smooth RGC, the parasol RGCs' sampling density of presynaptic bipolar cells was much higher, both in terms of the number of bipolar cells and the number of synapses per bipolar cell.

### Contributions of presynaptic bipolar cells to candidate hotspots

**Figure 8.**
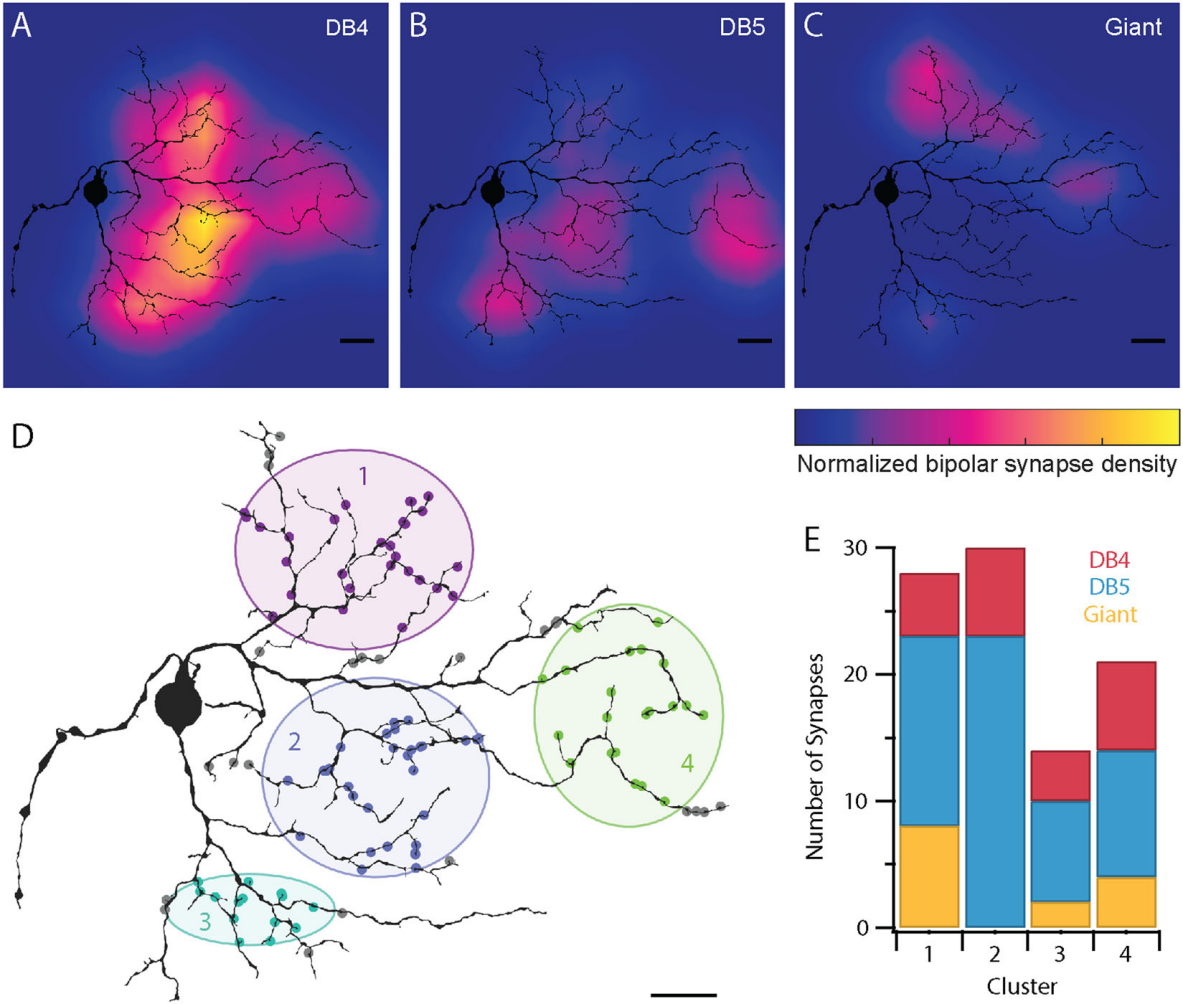
Density maps of the smooth RGC’s synaptic input from each bipolar cell type: DB4 (***A***), DB5 (***B***), and giant (***C***). The color maps are normalized together to show the relative contributions across bipolar cell types. (***D***) The smooth RGC’s ribbon synapses clustered by a mixture of Gaussians model (see Materials and Methods). The shaded areas are bounded by a distance of 3 SDs. Synapses exceeding this distance from all four clusters (gray) are omitted from the analysis in ***E***. (***E***) The relative contributions of DB4, DB5, and giant bipolar cell synapses to each of the four clusters. Scale bar, 20 µm. Details of the model fit and numerical data for ***E*** are provided in Extended Data Table 8-1.

We next looked at the spatial distribution of synaptic input to the smooth RGC from each bipolar cell type. Figure 8, *A–C*, shows density maps of the input from each bipolar cell type. Comparison with Figure 3*A* indicates that DB5 bipolar cells provided the dominant contribution to the candidate hotspots. The bias of giant bipolar cell input to the upper portion of the dendritic field in Figure 8*C* was unexpected; however, some of the bottom dendrites were clipped by the edge of the volume (Fig. 1*A*) so additional giant bipolar cell input in that region cannot be ruled out.

10.1523/ENEURO.0280-23.2023.t8-1 Extended Data Table 8-1. Mixture of Gaussians model fit for the four clusters within the smooth monostratified RGC's ribbon synapses. The centroid values are the X and Y coordinates within the EM volume in microns where 0, 0 is the bottom left corner. Download Table 8-1, DOCX file.

To obtain a more precise account of the contributions of DB4, DB5, and giant bipolar cells to individual hotspots, we estimated the location and spatial extent of the bipolar cell clusters by fitting the *x* and *y* coordinates of the ribbon synapses to a mixture of Gaussians model (Fig. 8*D*; see Materials and Methods). Consistent with the four clear hotspots seen in Figure 3*A*, a four-component model provided the best account of the underlying data. While the resulting model provides a basis for quantifying the contributions of each bipolar cell type to the candidate hotspots within the smooth RGC's dendritic field, the exact dimensions of the four components (Extended Data Table 8-1) are likely to be different from physiological measurements of hotspots. The important distinction to note is that the model is based on the locations of bipolar cell synapses rather than the cone inputs to each bipolar cell.

We next investigated the relative contributions of DB4, DB5, and giant bipolar cells to each of the candidate hotspots. Each cluster received input from multiple bipolar cell types (Fig. 8*E*), supporting the hypothesis that hotspots are the result of input from multiple excitatory cell types (Rhoades et al., 2019). As expected from Figures 4*E* and 8*C*, giant bipolar cell input varied the most between clusters. The variability in the relative contributions of each bipolar cell type to the four hotspots predicts that the response properties of each hotspot may vary as well.

### Relationship between parasol RGCs and candidate hotspots

A surprising feature of the smooth RGC's receptive field structure is that the hotspots and coolspots appear to coarsely align with the receptive fields of simultaneously-recorded parasol RGCs (Rhoades et al., 2019). We were curious whether a similar alignment is present for the three parasol RGCs in our study that were located within the smooth monostratified RGC's dendritic field.

**Figure 9.**
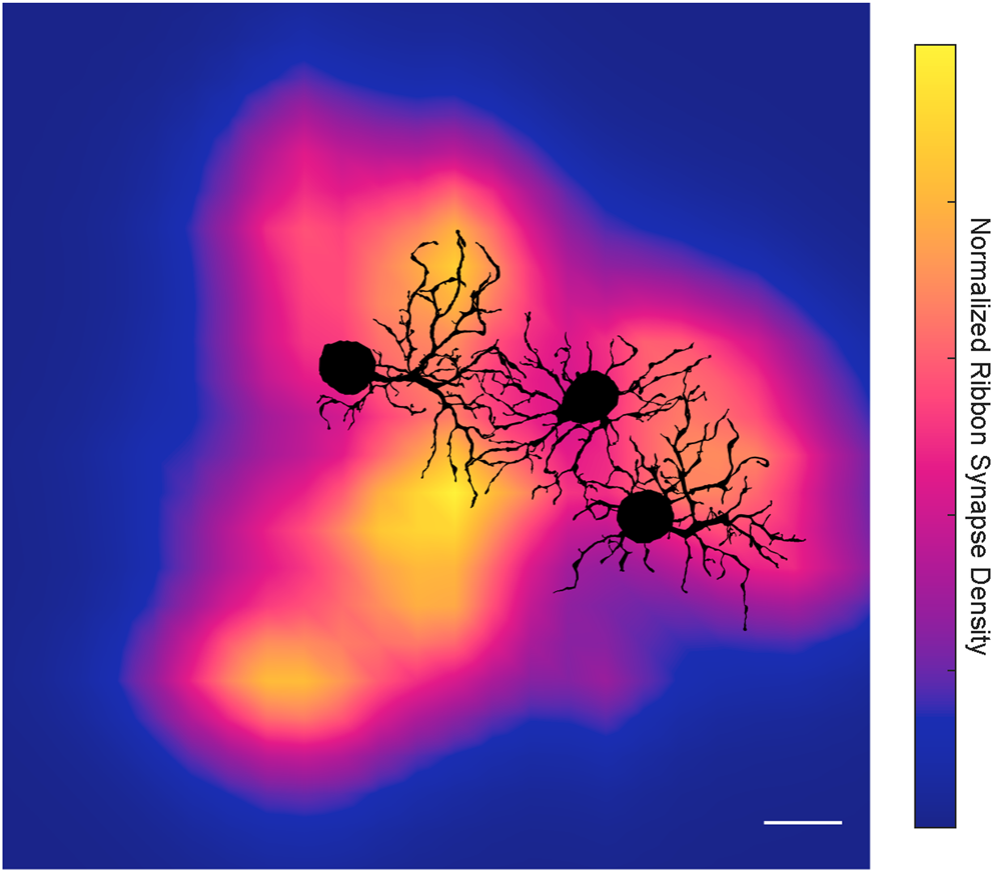
Relationship between the clusters of bipolar cell input to the smooth RGC and the dendritic fields of parasol RGCs. The center parasol falls within a gap in the bipolar cell input while the rightmost parasol RGC borders on a hotspot with dendrites extending toward both a hotspot and a coolspot. The left parasol RGC is located within a coolspot but has dendrites within two adjacent hotspots. The density map is from the second image on the top row of Figure 3*A*. Scale bar, 20 µm.

Figure 9 shows the parasol RGC's dendritic fields over the ribbon synapse density map from Figure 3*A*. The center parasol and most of its dendrites are positioned in a coolspot. The parasol RGC on the left and its primary dendrites are located within a coolspot, but many of its more distal dendrites extend to two adjacent hotspots. The rightmost parasol RGC and its primary dendrite border on a hotspot with dendrites extending toward both a hotspot and a coolspot. Importantly, two features of our anatomical analysis complicate comparisons with physiological measurements of smooth RGC hotspots and parasol RGC receptive fields. First, the physiological receptive fields represent 2D Gaussian fits with the edge drawn where sensitivity falls to 20% of the maximum (Rhoades et al., 2019), which may not extend to the edges of the underlying dendritic fields. Second, the density map in Figure 9 is based on the locations of bipolar cell synapses rather than the cone input to the underlying presynaptic bipolar cells. As such, our results are not inconsistent with the alignment reported physiologically, where the majority of parasol receptive fields were located within hotspots and coolspots, with only the outer edges impinging on opposing regions.

**Figure 10.**
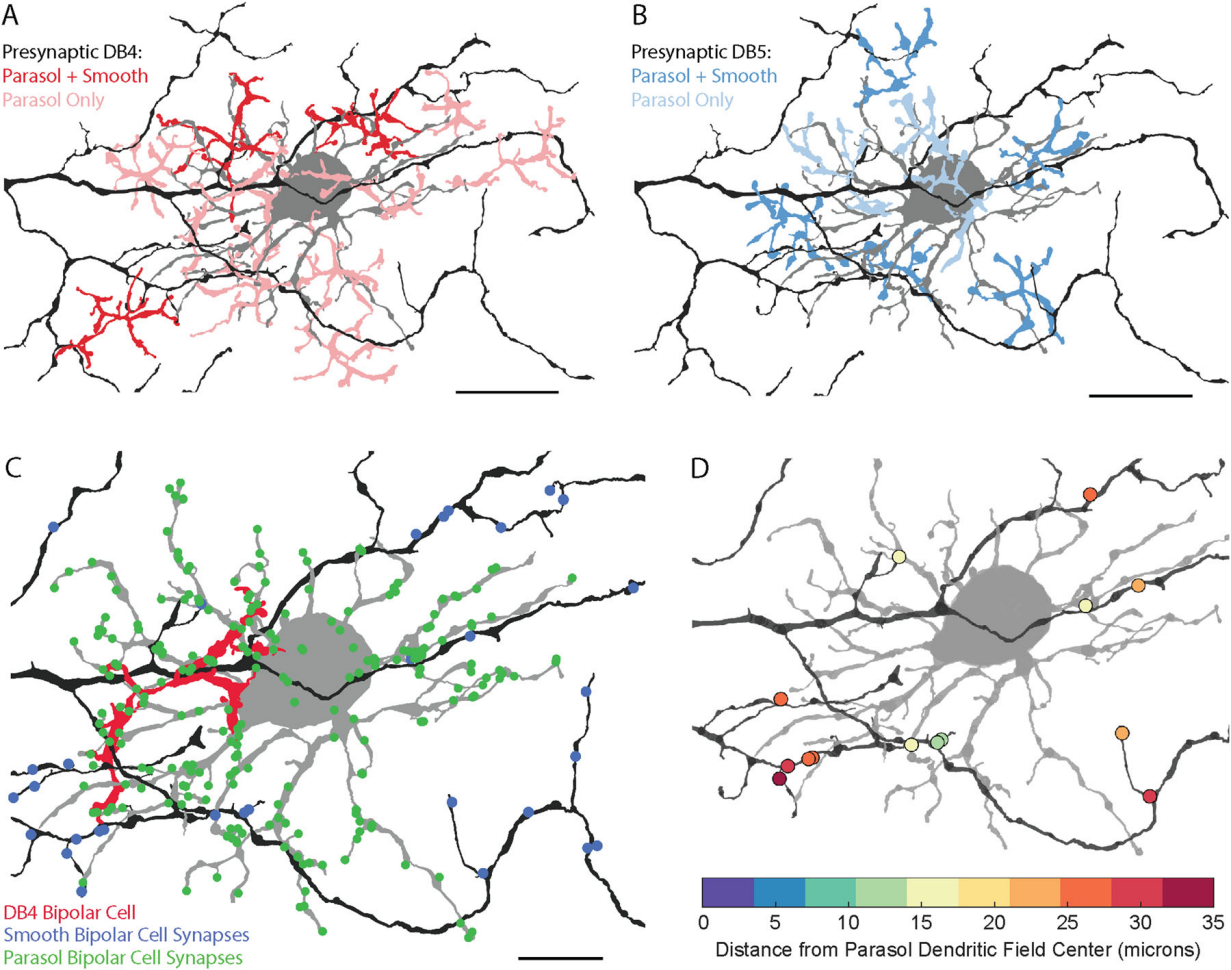
***A–B***, DB4 and DB5 bipolar cell input to the parasol RGC overlapping a candidate coolspot in the smooth RGC’s dendritic field. ***C***, Bipolar cell synapses to a parasol RGC within a coolspot in the smooth RGC’s dendritic field. The parasol (gray) receives dense bipolar cell input in a region where the smooth RGC receives very little bipolar cell input. A DB4 bipolar cell (red) provided 33 synapses to the parasol RGC but none to the nearby smooth RGC dendrites. EM micrographs showing this bipolar cell’s input to the parasol RGC and an adjacent parasol RGC near a smooth RGC dendrite are included in Extended Data Figure 10-1. For comparison, EM micrographs of bipolar cell input to the smooth RGC within a hotspot region are shown in Extended Data Figure 10-2. ***D***, Locations of bipolar cell synapses to the smooth RGC that fall within the dendritic field of the parasol RGC. The markers are colored by their distance from the parasol RGC’s dendritic field center. Scale bar, 20 µm.

To better understand the origins of the gaps in the smooth RGC's bipolar cell input, we focused on the coolspot region within the smooth RGC's dendritic field that overlapped the center parasol RGC in Figure 9. This parasol RGC received extensive bipolar cell input (Fig. 6), even though the dendrites of the overlapping smooth RGC did not. In total, only 11 of the 44 bipolar cells presynaptic to the parasol RGC also contacted the smooth RGC (5/33 DB5, 3/24 DB4, and 3/3 giant bipolar cells). Figure 10, *A* and *B*, shows parasol RGC's presynaptic DB4 and DB5 bipolar cells color-coded by whether they also synapsed on the smooth RGC. The parasol RGC sampled from a near-complete mosaic of DB4 and DB5 bipolar cells within its dendritic field. The few bipolar cells that also synapsed on the smooth RGC were largely located along the distal dendrites of the parasol RGC. Most DB4 and all DB5 bipolar cell axon terminals in the center of the parasol RGC's dendritic field provided no input to the smooth RGC.

The bipolar cells providing the most input to the parasol RGC (those within the center of the dendritic field) provided no input to the smooth RGC, despite their overlapping and co-stratifying dendrites (Fig. 10*A*,*B*). For example, the DB4 bipolar cell providing the most synaptic input to the parasol RGC (33 synapses) did not contact the smooth RGC (Fig. 10*C*). While the smooth RGC's dendritic field is sparser, this alone cannot explain the lack of bipolar cell input in the regions overlapping the parasol RGC's dendritic fields as the smooth RGC's dendrites were often in close proximity to the bipolar cell processes (Fig. 10*C* and Extended Data Fig. 10-1).

In total, the smooth RGC received 14 bipolar cell synapses within the region overlapping the parasol RGC's dendritic field, most of which were biased toward the parasol RGC's distal dendrites, despite the smooth RGC having dendrites overlying the parasol RGC's soma and primary dendrites. For each bipolar synapse onto the smooth RGC, we calculated its distance from the parasol RGC's dendritic field center (Fig. 10*D*) and found the average distance was 22.51 ± 6.68 μm (*n* = 14). The closest smooth RGC synapse to the parasol RGC's dendritic field center was 12.8 μm away.

The smooth RGC's morphology within the coolspot region was also notable. This area corresponded to a region with low dendrite density in Figure 3*A*. In other areas of the smooth RGC's dendritic field, bipolar cell input was commonly seen at the end of small branches extending from a larger dendrite. These short branches appeared to target bipolar cells because they ended abruptly after receiving ribbon synaptic input (Extended Data Fig. 10-2). Taken together, the smooth RGC's structure and synaptic input indicate that some parasol RGCs are located in potential coolspots and demonstrate that, within these regions, smooth RGC dendrites avoid bipolar cell input. Understanding how the smooth RGC's clustered bipolar cell input arises in development and whether the dendritic fields of overlapping parasol RGCs could serve as a cue for hotspot/coolspot formation will be an interesting direction for future research.

## Discussion

We understand a great deal about the most common primate RGCs, but very little about the many rarer RGC types which are difficult to reliably find and systematically investigate with standard electrophysiology techniques. The connectomics approach employed here provides a tractable alternative for making progress on the underlying mechanisms of rarer retinal neurons that complements physiology by explaining past results and guiding future experiments (Denk et al., 2012; Patterson et al., 2022). Here, we provide the first account of the synaptic inputs to ON smooth RGCs, which make up just 3% of the retinal output (Grünert and Martin, 2021), and compare their circuitry with the well-studied ON parasol RGCs.

The parasol and smooth RGCs provide an interesting case study in neuronal computation as they derive distinct spatial receptive fields and response properties from a common set of synaptic inputs. First, the smooth RGCs received a greater proportion of amacrine cell input compared with parasol RGCs. Second, the relative amounts of input from each co-stratifying bipolar cell type differed. Third, parasol RGCs densely sampled presynaptic bipolar cells, while the smooth RGC received just 1–2 synapses from most bipolar cells. Fourth, unlike the parasol RGCs, bipolar cell input to the smooth RGC formed several distinct clusters comparable with the receptive field hotspots identified with electrophysiology.

A striking feature of the smooth RGCs' receptive field is the isolated regions of sensitivity (hotspots) surrounded by regions of low sensitivity (coolspots). Our data reveal an underlying mechanism in the clustering of synaptic input from bipolar cells (Figs. 3*A*, 4*B*, 8*D*). The smooth RGC's morphology played a supporting role in establishing the hotspots through the soma placement and sparse dendritic branching. Multiple bipolar cell types contributed to each hotspot, ruling out the possibility that the large axon terminals of giant bipolar cells are responsible for the smooth RGC's hotspots. Instead, DB5 bipolar cells made the dominant contribution, providing 48–83% of the bipolar cell input to each cluster in Figure 8*E*.

While the smooth RGC's hotspots are unique among known primate RGCs, similar spatial heterogeneity is a common feature of mammalian Y-cells (Thibos and Levick, 1983; Soodak et al., 1991; Schwartz et al., 2012). These spatially localized regions of sensitivity have been explained by the locations of excitatory bipolar cell input to the Y-cell and, to a lesser extent, the pattern of dendritic branching (Freed et al., 1992; Brown et al., 2000). Y-cells (also called alpha cells) have transient responses, larger receptive fields, and nonlinear spatial integration (Enroth-Cugell and Robson, 1966; Peichl et al., 1987). The parasol RGC's status as a Y-cells is debated (Dreher et al., 1976; Kaplan and Shapley, 1982; Derrington and Lennie, 1984; White et al., 2002; Crook et al., 2008b), while the smooth RGCs are proposed to provide a less ambiguous Y-cell counterpart (Petrusca et al., 2007). While the X/Y/W classification system has largely been abandoned for systems that better account for RGC diversity (Baden et al., 2016; Goetz et al., 2022), Y-cells remain one of the best studied mammalian RGC classes. The value in connecting this work to primate RGCs is the opportunity to draw on this extensive literature which can provide a valuable reference for understanding rarer primate RGCs.

The Y-cell's characteristic nonlinear spatial integration arises through nonlinear subunits established by the rectified outputs of presynaptic bipolar cells (Hochstein and Shapley, 1976; Demb et al., 2001a; Schwartz and Rieke, 2011). These nonlinear subunits confer sensitivity to spatial structure smaller than the RGC's linear receptive field. In parasol RGCs, diffuse bipolar cells are thought to be the basis for these nonlinearities (Puthussery et al., 2013). The DB5 and DB4 bipolar cell input to the smooth RGCs (Figs. 4*C*, 4*D*, 8*E*) would be expected to create similar subunits within each hotspot. Accordingly, nonlinear spatial integration is observed at two spatial scales—hotspots and the rectified outputs of the bipolar cells driving each hotspot (Petrusca et al., 2007; Crook et al., 2008a; Rhoades et al., 2019). Of the two, the lower-resolution hotspots appear to be the dominant nonlinear subunit (Rhoades et al., 2019). The differences in sampling density of presynaptic bipolar cells in Figure 7 may help explain this observation. Parasol RGCs received dense input from most presynaptic bipolar cells while the smooth RGC typically collected just a few synapses per bipolar cell. By minimizing the contributions from each presynaptic bipolar cell, the smooth RGC may limit the strength of the resulting higher-resolution subunits within each hotspot.

Parasol and smooth RGCs have been proposed to serve as complementary pathways differing only in their spatial tuning (Crook et al., 2008a), consistent with the classic spatiotemporal frequency channels hypothesis for the early visual system (Campbell and Robson, 1968; Pollen et al., 1971; Barlow, 1979). However, the discovery of the smooth RGC's hotspots has re-opened the key question of why primates have smooth RGCs. While the smooth RGC's hotspots do perform nonlinear spatial integration at a lower resolution than the parasol RGCs, they differ in several key ways from the parasol RGC's bipolar cell subunits. First, the hotspots are spatially segregated while the diffuse bipolar cell input to parasol RGCs overlaps, a distinction that can be appreciated by comparing the spacing between the clusters in Figure 8*D* to the tight tiling of the parasol RGC's presynaptic bipolar cells in Figure 6. Second, while the bipolar cell subunits are largely homogeneous and often synchronized through gap junctions (Jacoby and Marshak, 1999; Dacey et al., 2000; Manookin et al., 2018), the smooth RGC's hotspots differ in more than just their spatial position within the receptive field. Surprisingly, different extracellularly-recorded action potential waveforms arise when stimulating each hotspot individually, suggesting that each hotspot plays a distinct role in spike generation and may even have different visual response properties (Diamond, 2019; Rhoades et al., 2019). The variable bipolar cell input to each hotspot (Fig. 8*E*) could contribute to these differences while the dominance of amacrine cell input between hotspots could aid in electrically isolating these regions. However, a full account of the hotspots' unique responses will require a better understanding of the underlying biophysics. Connectomic reconstructions such as ours can provide a valuable basis for realistic biophysical models exploring this question (Koch et al., 1982; Freed et al., 1992).

ON midget bipolar cells were the only co-stratifying bipolar cell type that did not contact the smooth RGCs, but they provided a quarter of the parasol RGCs' bipolar cell input (Figs. 5*B*, 6*E*). While both parasol and smooth RGCs have transient initial responses, only the parasol RGCs maintain a sustained firing rate during long contrast modulations (Petrusca et al., 2007), which could reflect the contributions of presynaptic midget bipolar cells. The parasol RGCs' substantial midget bipolar cell input is notable in the central retina where the midget/parvocellular circuitry establishes the specialized primate “private line” pathway (Kolb and Marshak, 2003).

ON midget bipolar cells were not the only neuron with primate-specific specializations to prefer parasol RGCs. DB4 bipolar cells provided over half of the parasol RGCs' bipolar cell input but only a quarter of the smooth RGC's input (Fig. 5). Unlike transient bipolar cells in non-primate mammalian retinas, DB4 bipolar cell axons have active conductances and can generate action potentials under some conditions which enhance their transient excitation of postsynaptic RGCs (Puthussery et al., 2013). Far less is known about the physiology of the DB5 bipolar cells that provide the dominant input to the smooth RGCs; however, their stratification within the inner retina suggests that their responses are more sustained than those of the DB4 bipolar cell (Rodieck, 1998; Euler et al., 2014). If so, the rapid, transient responses reported for smooth RGCs (Petrusca et al., 2007) are likely to be shaped by amacrine cell input rather than inherited from presynaptic bipolar cells.

Although we chose to focus on the ON pathway, our major findings related to the diffuse bipolar cells are likely mirrored in diffuse OFF bipolar cell input to the OFF smooth RGC's hotspots. However, both parasol and smooth RGCs received synaptic input from giant bipolar cells (Fig. 5), which have no known OFF bipolar cell counterpart. As a result, the giant bipolar cell's contributions may lead to yet undiscovered asymmetries between the ON and OFF smooth monostratified RGCs. Giant bipolar cells are unique among primate bipolar cells in that they contact a sparse population of cones and rods over a large area of the photoreceptor mosaic (Tsukamoto and Omi, 2016). Little is known about the giant bipolar cell's response properties (Puthussery et al., 2013), and their role in retinal processing remains an open question. Our identification of two RGC types receiving giant bipolar cell input provides a first step toward understanding their contributions to vision.

Many of the differences between the parasol and smooth RGC's circuitry identified here do not have clear links to know physiological differences, indicating we may have much to learn about both RGC types. Primate RGCs were long thought to lack the complex response properties found in other species, and the literature is dominated by artificial stimuli designed for simple center–surround receptive fields. These stimuli are valuable for RGC classification because they each isolate specific underlying mechanisms and permit analysis with simplified quantitative models. By design, these stimuli obscure additional complexity within underlying receptive fields. For example, in studies using grating stimuli, the smooth RGC's hotspots were not apparent (Petrusca et al., 2007; Crook et al., 2008a) and, in many cases, smooth and parasol RGCs were difficult to distinguish (Derrington and Lennie, 1984). Exploring a broader range of stimuli is likely to reveal more of the ways in which the differences in synaptic input reported here distinguish the parasol and smooth RGCs response properties. For example, stimuli matching the statistics of natural scenes engage more underlying mechanisms and their often-nonlinear interactions (Turner et al., 2019) and have recently revealed new insight into parasol RGCs (Turner and Rieke, 2016; Freedland and Rieke, 2022). A second potential avenue to explore will be stimuli tailored toward specific downstream visual functions.

A straightforward explanation for the differences between ON smooth monostratified and ON parasol RGC circuitry reported here is that they are uniquely tailored for distinct roles in vision. While our results help explain *how* the smooth RGC's hotspots arise, the larger question of *why* smooth RGCs have such unexpected receptive fields remains a mystery. Nonlinear spatial integration in mammalian Y-cells has been explored in the context of visual functions classically attributed to the primate cortex such as second-order motion, defocus and texture sensitivity (Demb et al., 2001b; Schwartz et al., 2012). The smooth RGC's hotspots are difficult to reconcile with standard models of primate visual processing that limit the goal of retinal computations to faithful and efficient transmission of visual information to the cortex. Because the smooth monostratified RGCs challenge our current models of primate vision, efforts to better understand them may ultimately lead to a better understanding of the primate retina's role in visual perception and behavior.

## Synthesis

Reviewing Editor: Nicholas J. Priebe, University of Texas at Austin Decisions are customarily a result of the Reviewing Editor and the peer reviewers coming together and discussing their recommendations until a consensus is reached. When revisions are invited, a fact-based synthesis statement explaining their decision and outlining what is needed to prepare a revision will be listed below. The following reviewer(s) agreed to reveal their identity: Joshua Singer. Note: If this manuscript was transferred from JNeurosci and a decision was made to accept the manuscript without peer review, a brief statement to this effect will instead be what is listed below.

Two reviewers evaluated the revision of the manuscript "Synaptic origins of the complex receptive field structure in primate smooth monostratified retinal ganglion cells" and both agree that the study provides valuable information about how distinct receptive field profiles emerge from retinal ganglion cells classes positioned to receive similar inputs. Both agreed that the experimental results are of high quality, but they raised issues related to both the data presentation and the text of the manuscript. Overall, the reviewers felt that the manuscript needs a careful and thorough revision. These issues are detailed below, which you should find useful in revising your manuscript, if you decide to resubmit it to eNeuro or another journal.

Major Issues:

1. Data presentation

A) Additional EM images.

Both reviewers agreed that additional images would strengthen the manuscript. While it is the case that readers will be able to visualize the raw data (through the use of the Viking software), both reviewers agreed that additional images describing showing additional examples of amacrine cell synapses as well as sequential sections of amacrine and bipolar cell synapses (as in Figure S1 from Graydon et al. 2018 and Figure S1 from Grimes et al. 2022 ) will enhance the manuscript.

B) Mismatches between figures

Please check Figure 2A. Some of the peripheral dendrites on the right-hand side do not show synaptic input at all, whereas Figure 3 and Figure 6 show that these dendrites are contacted by bipolar cells. Moreover, the legend to Figure 2 mentions black arrowheads but there are no black arrowheads in the Figure. Similarly, please compare Figure 3 and 6, with respect to the GB input.

2. Text clarifications

A) The authors state that "Table 1 has been deleted and the data has been updated or added to the text." Did the text include the dendritic field size for the smooth mono and parasol cells?

B) The section "Relationship between parasol RGCs and bipolar input to smooth RGCs" needs a better header and should be rewritten for clarity. For example, Line 320 ff. After reading this paragraph multiple times, the understanding is: In the regions where the dendrites of parasol and SM cells overlapped, parasol cells received extensive bipolar input whereas SM cells only received little bipolar input. However, looking at the orange oval in Figure 9A the numbers of green dots (n=12 and n=9 which equate to the number of ribbon synapses, respectively onto the two parasol cells) and blue dots (n=9 ribbon synapses onto the smooth monostratified cell) are not very different. The differences mentioned with respect to the proximal versus the distal dendritic field are not clear. The triangle and the oval occupy areas of different sizes.

C) The following information for the two cell types should be provided in the text: How many regions of bipolar clusters were observed, how large were these areas and how many bipolar/amacrine synapses were counted in these regions?

D) Legend to Fig. 9B-D. The legend provided is confusing. It could be simplified to something like this: Two DB4 axon terminals (bright pink) overlap with the dendrites of both parasol and smooth monostratified cell types but contact only parasol cells. These DB4 axon terminals provided the highest number of synapses onto the respective parasol cell (pink dots).

E) There is only a very brief reference of Figure 10 in the manuscript.

F) Line 339ff. The two last sentences of the result section seem to contradict each other. "... a cluster of bipolar cell synapses onto the smooth RGC is seen in the region where the distal dendrites of parasol cells overlap, ..." "these results suggest ... that ... within this region, smooth RGC dendrites avoid bipolar cell input."

Minor Issues:

1. Calculating the dendritic field diameters of all cells from your dendritic area measurements, would allow a better direct comparison with the three previous studies on smooth monostratified cells cited in the manuscript (Petrusca et al.; Rhoades et al., Crook et al.).

2. Line 281: should read "...a cluster ... [was] evident ..."

3. Line 287: "The parasol RGCs [also] received ..." suggest deleting "also"

4. Line 330: ... surrounded by amacrine input (Fig. 9A orange triangle): this is not visible in the figure.

5. Line 397: "... [also] received larger proportion of their total synaptic input ... " please add "compared to ON parasol cells" and delete "also".

6. Line 327: "...the few bipolar synapses onto the smooth RGC that did overlap..." consider revising.

7. Please provide a key for Figure 9.

8. Extended tables: instead of "midget" use IMB (for invaginating midget bipolar cell)

9. Figure 2: please add parasol cell ID to the legend.

## Author Response

Dear Dr. Priebe,

We thank the reviewers for their thoughtful and constructive comments. We have revised the manuscript in response to the reviewers' comments as detailed below, with our responses indented in italics.

---------------------------------------------

Synthesis of Reviews:

Computational Neuroscience Model Code Accessibility Comments for Author (Required):

N/A

Synthesis Statement for Author (Required):

SYNTHESIS

Two reviewers evaluated the revision of the manuscript "Synaptic origins of the complex receptive field structure in primate smooth monostratified retinal ganglion cells" and both agree that the study provides valuable information about how distinct receptive field profiles emerge from retinal ganglion cells classes positioned to receive similar inputs. Both agreed that the experimental results are of high quality, but they raised issues related to both the data presentation and the text of the manuscript. Overall, the reviewers felt that the manuscript needs a careful and thorough revision. These issues are detailed below, which you should find useful in revising your manuscript, if you decide to resubmit it to eNeuro or another journal.

Major Issues:

1. Data presentation

A) Additional EM images.

Both reviewers agreed that additional images would strengthen the manuscript. While it is the case that readers will be able to visualize the raw data (through the use of the Viking software), both reviewers agreed that additional images describing showing additional examples of amacrine cell synapses as well as sequential sections of amacrine and bipolar cell synapses (as in Figure S1 from Graydon et al. 2018 and Figure S1 from Grimes et al. 2022 ) will enhance the manuscript.

We have included two additional figures with EM images, Extended Data Figures 10-1 and 10-2. These figures include sequential sections of amacrine and bipolar synapses. Prior EM Figures 4 and 7 have been renumbered as Extended Data Figures 4-1 and 6-1, respectively.

B) Mismatches between figures

Please check Figure 2A. Some of the peripheral dendrites on the right-hand side do not show synaptic input at all, whereas Figure 3 and Figure 6 show that these dendrites are contacted by bipolar cells. Moreover, the legend to Figure 2 mentions black arrowheads but there are no black arrowheads in the Figure. Similarly, please compare Figure 3 and 6, with respect to the GB input.

We thank the reviewers for catching these errors. All figures and tables have been rechecked and corrected.

2. Text clarifications

A) The authors state that "Table 1 has been deleted and the data has been updated or added to the text." Did the text include the dendritic field size for the smooth mono and parasol cells?

We apologize for this oversight. As requested, the dendritic field area has been replaced with diameter, included in the second paragraph of the results and now added to Extended Data Table 1-1.

B) The section "Relationship between parasol RGCs and bipolar input to smooth RGCs" needs a better header and should be rewritten for clarity. For example, Line 320 ff. After reading this paragraph multiple times, the understanding is: In the regions where the dendrites of parasol and SM cells overlapped, parasol cells received extensive bipolar input whereas SM cells only received little bipolar input. However, looking at the orange oval in Figure 9A the numbers of green dots (n=12 and n=9 which equate to the number of ribbon synapses, respectively onto the two parasol cells) and blue dots (n=9 ribbon synapses onto the smooth monostratified cell) are not very different. The differences mentioned with respect to the proximal versus the distal dendritic field are not clear. The triangle and the oval occupy areas of different sizes.

The most substantial changes to the Results were in the final section where the reviewers raised several issues (Comments B-F). We agree that this section was confusing and the key points were difficult to discern in the figures. In the revised manuscript, we have taken a different approach to displaying the data such that it more effectively illustrates the relationship between the parasol RGCs and the hotspots and coolspots. Specifically, instead of relying entirely on images displaying synapses, we now also show density maps built from 2D histograms of the synapses (Figures 3, 8, and 9). This approach is now detailed in the Methods section "Quantification and visualization of bipolar cell clusters." Edits were made in the Results section to integrate the improved visualization and incorporate the quantification.

These changes also required modifications to the Discussion section to maintain flow and clarity. Many of the bolded changes in the Results and Discussion sections involved rewording text without changing the intent. In addition, the authors felt that previous discussion of Y-cells lacked context, so this section was removed from the introduction and expanded in the Discussion (4th and 5th paragraphs).

C) The following information for the two cell types should be provided in the text: How many regions of bipolar clusters were observed, how large were these areas and how many bipolar/amacrine synapses were counted in these regions?

This information is now included in Figures 3A and 8 and in the text associated with these figures.

D) Legend to Fig. 9B-D. The legend provided is confusing. It could be simplified to something like this: Two DB4 axon terminals (bright pink) overlap with the dendrites of both parasol and smooth monostratified cell types but contact only parasol cells. These DB4 axon terminals provided the highest number of synapses onto the respective parasol cell (pink dots).

E) There is only a very brief reference of Figure 10 in the manuscript.

Figures 9 and 10 of the original manuscript were provided to illustrate the relationship between the parasol cells and candidate hotspots/coolspots. These figures have now been replaced with the density map in the new Figure 9. The new Figure 10 includes parts of the previous Figures 9 and 10 in order to focus on a specific example of a coolspot (two paragraphs beginning "To better understand the origins...." on p19).

F) Line 339ff. The two last sentences of the result section seem to contradict each other. "... a cluster of bipolar cell synapses onto the smooth RGC is seen in the region where the distal dendrites of parasol cells overlap, ..." "these results suggest ... that ... within this region, smooth RGC dendrites avoid bipolar cell input."

This section has been rewritten and the key point in the last sentence has been clarified. The 2nd to last sentence was no longer relevant since it refered to a figure that has been removed.

Minor Issues:

1. Calculating the dendritic field diameters of all cells from your dendritic area measurements, would allow a better direct comparison with the three previous studies on smooth monostratified cells cited in the manuscript (Petrusca et al.; Rhoades et al., Crook et al.).

Dendritic field diamteres are now included (Extended Data Table 1-1).

2. Line 281: should read "...a cluster ... [was] evident ..."

This sentence has been rewritten.

3. Line 287: "The parasol RGCs [also] received ..." suggest deleting "also"

"also" has been deleted.

4. Line 330: ... surrounded by amacrine input (Fig. 9A orange triangle): this is not visible in the figure.

This figure has been replaced.

5. Line 397: "... [also] received larger proportion of their total synaptic input ... " please add "compared to ON parasol cells" and delete "also".

This has been added.

6. Line 327: "...the few bipolar synapses onto the smooth RGC that did overlap..." consider revising.

This section has been revised with the new figures.

7. Please provide a key for Figure 9.

Keys are now provided for all figures.

8. Extended tables: instead of "midget" use IMB (for invaginating midget bipolar cell)

Tables have been modified as suggested.

9. Figure 2: please add parasol cell ID to the legend.

This is specified in the methods and has also been added to the legend.
