# Supplementary figures and images for: Synaptic Origins of the Complex Receptive Field Structure in Primate Smooth Monostratified Retinal Ganglion Cells
Source: eNeuro. 2024 Jan 25;11(1):ENEURO.0280-23.2023. doi: 10.1523/ENEURO.0280-23.2023 (PMC11078106; doi:10.1523/ENEURO.0280-23.2023)

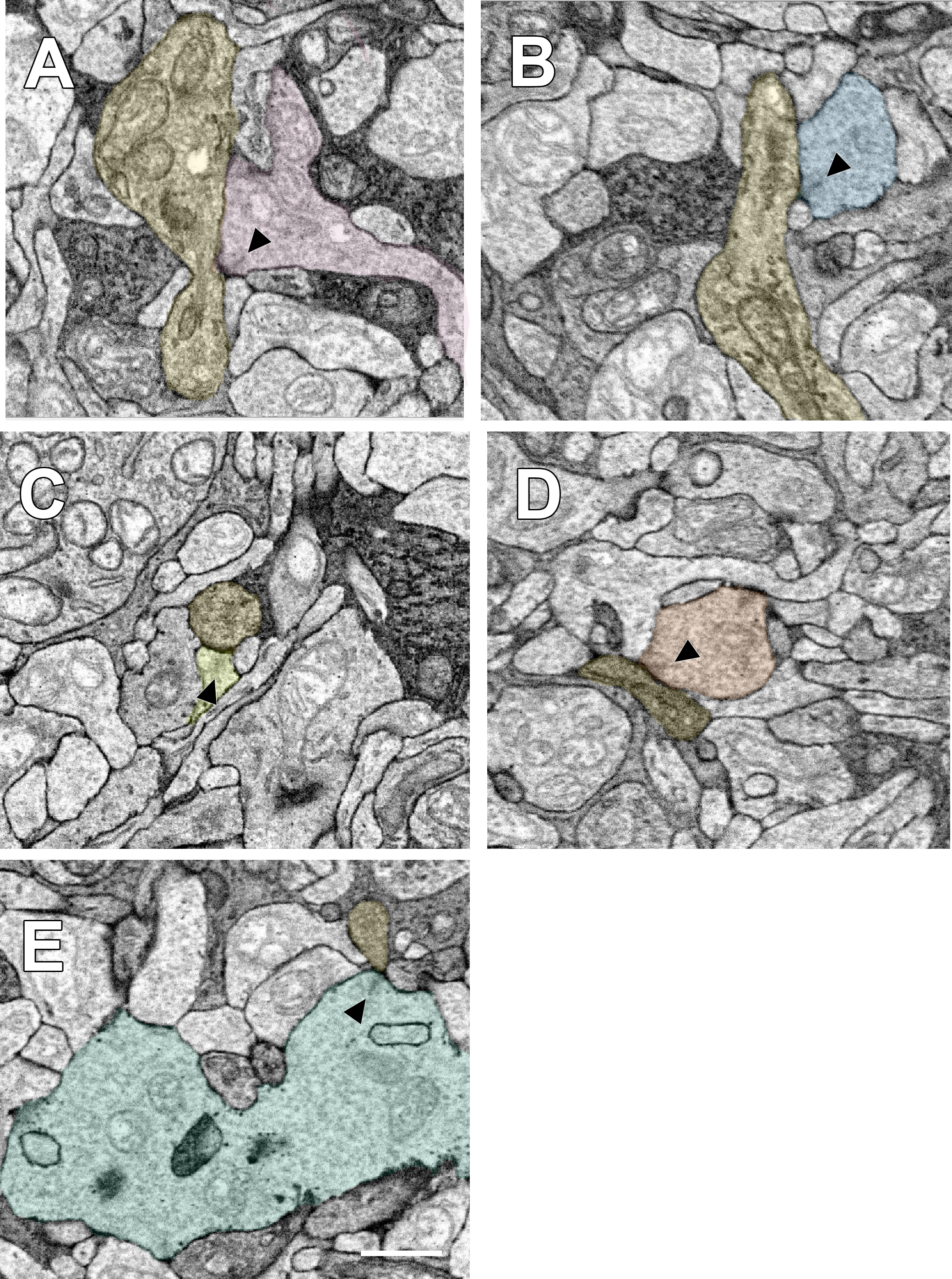

Supplement: Extended Data Figure 4-1. — Bipolar cell inputs to an ON smooth monostratified RGC (yellow). A. DB4 bipolar cell (pink), B. DB5 bipolar cell (blue), C. Giant bipolar cell (orange). Synaptic ribbons are indicated by black arrowheads. Scale bar = 1 μm. Download Figure 4-1, TIF file. [file eneuro-11-ENEURO.0280-23.2023-s001.tif]

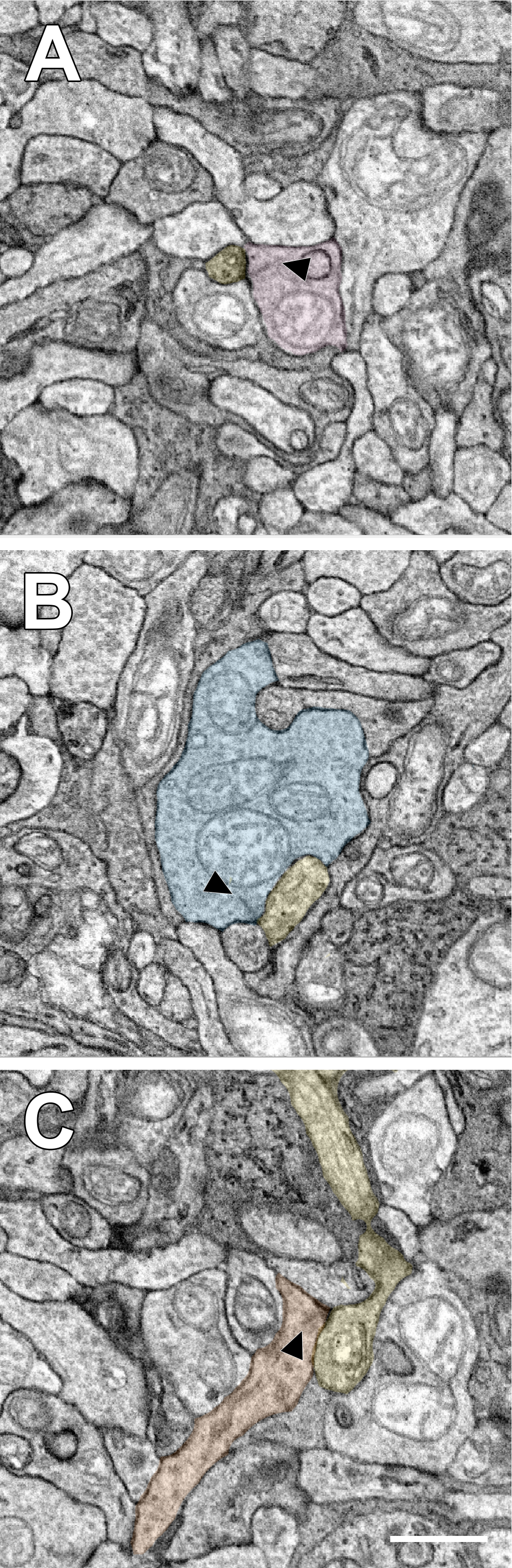

Supplement: Extended Data Figure 6-1. — Bipolar cell inputs to ON parasol RGC dendrites (dark yellow). A. DB4 bipolar cell (pink), B. DB5 bipolar cell (blue), C. DB6 bipolar cell (bright yellow), D. Giant bipolar cell (orange), D. ON midget bipolar cell (green). Synaptic ribbons are indicated by black arrowheads. Scale bar = 1 μm. Download Figure 6-1, TIF file. [file eneuro-11-ENEURO.0280-23.2023-s002.tif]

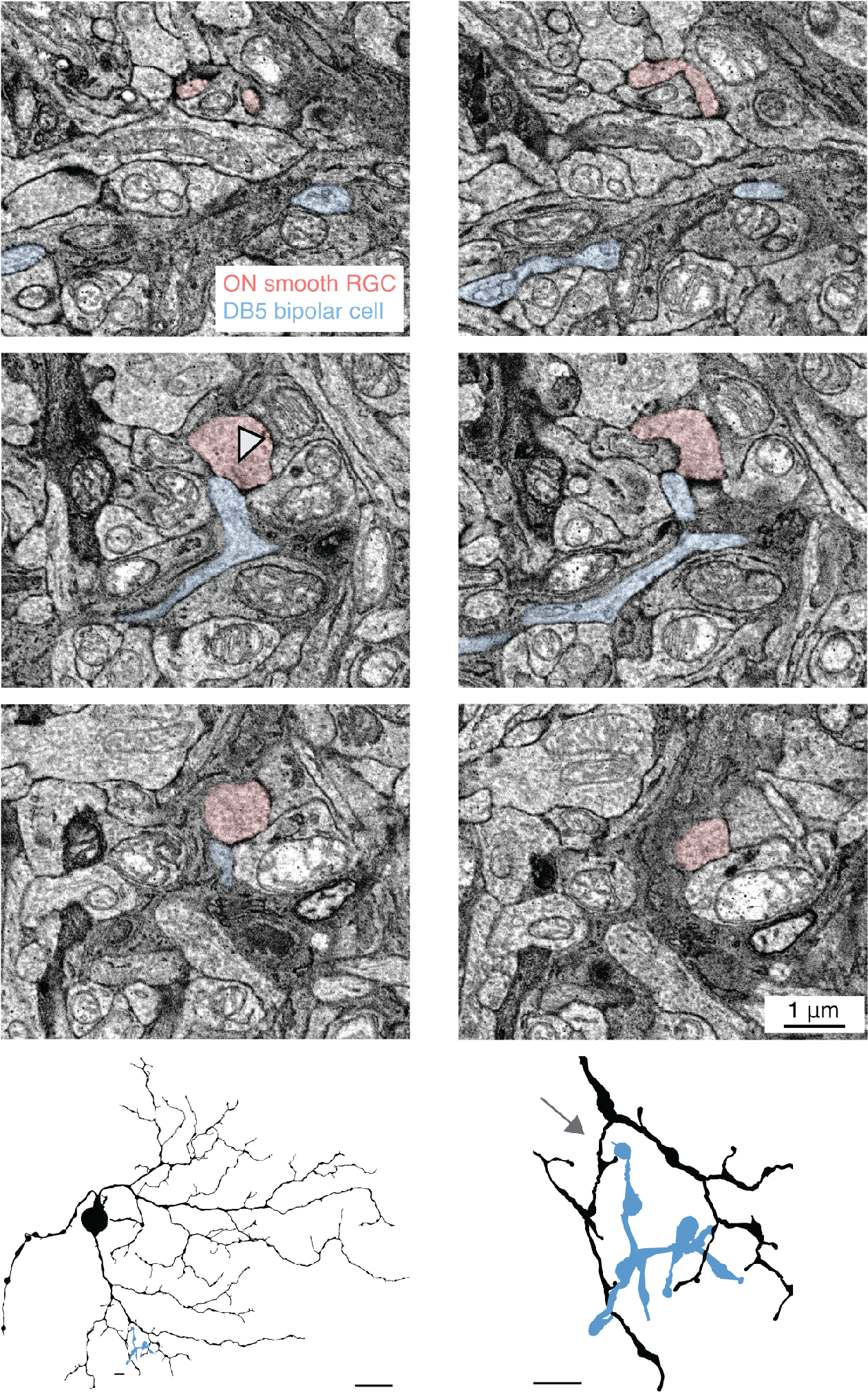

Supplement: Extended Data Figure 10-1. — Example synapses from a candidate coolspot within the smooth monostratified RGC's dendritic field shown in serial sections. Arrows mark DB4 synapses onto two parasols (5063 and 18269) in the 2nd and 4th rows. The smooth RGC receives no bipolar cell input. A representative amacrine cell synapse onto the smooth RGC is marked with an arrow in the 3rd row. Download Figure 10-1, TIF file. [file eneuro-11-ENEURO.0280-23.2023-s003.tif]

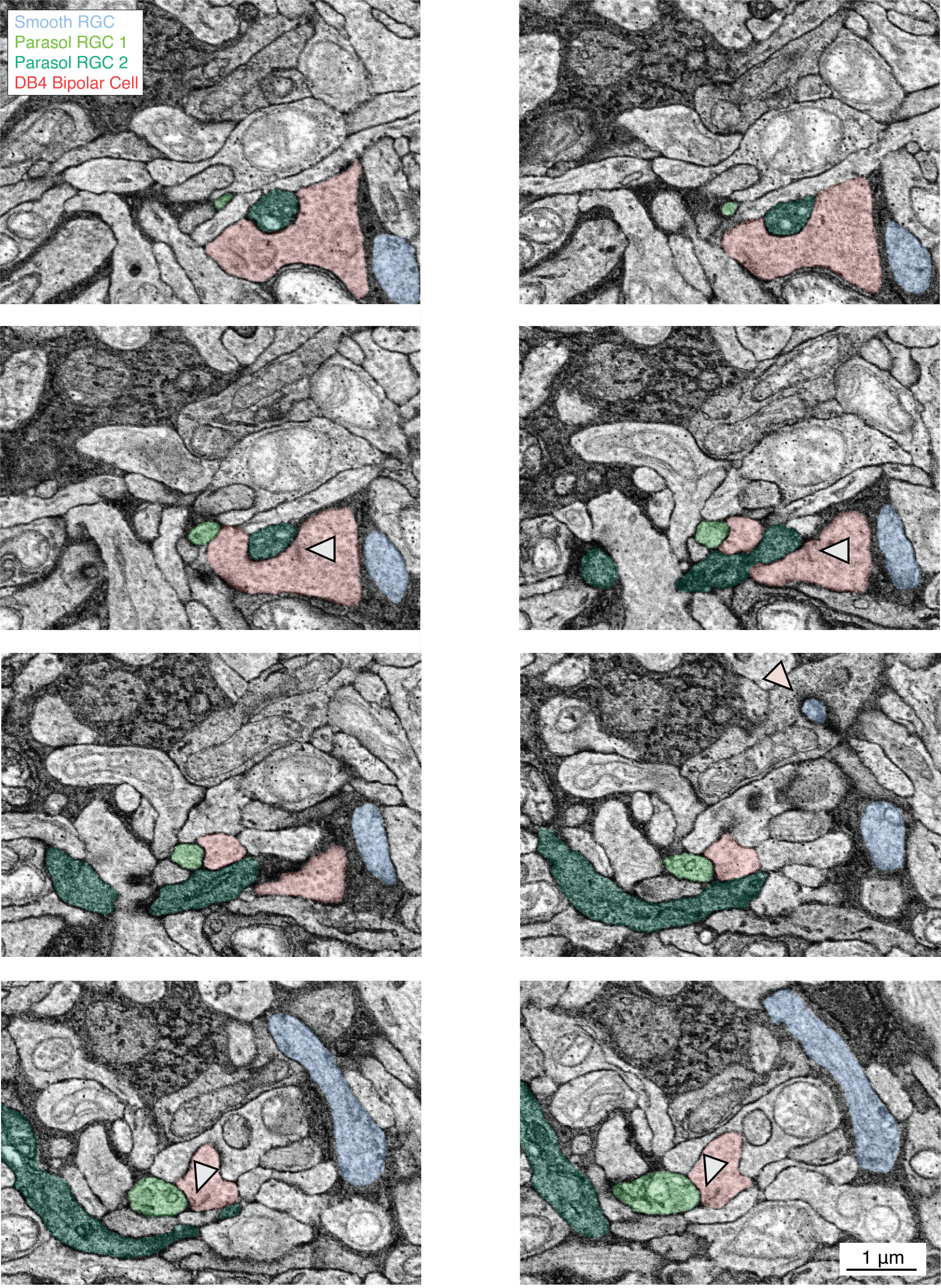

Supplement: Extended Data Figure 10-2. — An example synapse from a DB5 bipolar cell onto the smooth RGC within a hotspot region of the dendritic field shown in serial sections. A short off-shoot branch from the smooth RGC extends towards the bipolar cell and ends abruptly after receiving input from a ribbon synapse (arrow). The bottom row shows the location of the diffuse bipolar cell within the smooth RGC's dendritic field. Bottom left scale bar = 20 μm, bottom right scale bar = 5 μm. Download Figure 10-2, TIF file. [file eneuro-11-ENEURO.0280-23.2023-s004.tif]
