# Supplementary material for: Synaptic Origins of the Complex Receptive Field Structure in Primate Smooth Monostratified Retinal Ganglion Cells
Source: eNeuro. 2024 Jan 25;11(1):ENEURO.0280-23.2023. doi: 10.1523/ENEURO.0280-23.2023 (PMC11078106; doi:10.1523/ENEURO.0280-23.2023)
Supplement: Extended Data Table 1-1. — Dendritic field diameters for parasol and smooth monostratified RGCs. Download Table 1-1, DOCX file. [file eneuro-11-ENEURO.0280-23.2023-s006.docx]

Extended Data Table 1-1. Dendritic field diameters for smooth and parasol RGCs.

| Neuron | Dendritic Field Diameter (μm) |
| --- | --- |
| Smooth RGC 1321 | 144.098 |
| Smooth RGC 7889 | 161.913 |
| Parasol RGC 5035 | 58.188 |
| Parasol RGC 5063 | 55.434 |
| Parasol RGC 5370 | 54.154 |
| Parasol RGC 18269 | 58.810 |
| Parasol RGC 21392 | 58.355 |
