# Supplementary material for: Synaptic Origins of the Complex Receptive Field Structure in Primate Smooth Monostratified Retinal Ganglion Cells
Source: eNeuro. 2024 Jan 25;11(1):ENEURO.0280-23.2023. doi: 10.1523/ENEURO.0280-23.2023 (PMC11078106; doi:10.1523/ENEURO.0280-23.2023)
Supplement: Extended Data Table 5-1. — Total presynaptic bipolar cells for smooth monostratified RGC (1321) and three parasol RGCs (5063, 5370, 18269) by type. IMB stands for invaginating ("ON") midget bipolar cell. Download Table 5-1, DOCX file. [file eneuro-11-ENEURO.0280-23.2023-s007.docx]

Extended Data Table 5-1. Total bipolar cell input to parasol and smooth RGCs by type

| Neuron | DB4 | DB5 | DB6 | Giant | IMB | Unknown | Total |
| --- | --- | --- | --- | --- | --- | --- | --- |
| Smooth 1321 | 24 (34.8%) | 33 (47.8%) | 0 (0%) | 6 (8.7%) | 0 (0%) | 6 (8.7%) | 69 |
| Parasol 5063 | 12 (30.0%) | 5 (12.5%) | 0 (0%) | 1 (2.5%) | 20 (50.0%) | 2 (5.0%) | 40 |
| Parasol 5370 | 12 (31.6%) | 10 (26.3%) | 0 (0%) | 1 (2.6%) | 13 (34.2%) | 2 (5.3%) | 38 |
| Parasol 18269 | 11 (27.3%) | 8 (18.2%) | 1 (2.3%) | 3 (6.8%) | 16 (36.4%) | 4 (9.1%) | 43 |
