# Supplementary material for: Synaptic Origins of the Complex Receptive Field Structure in Primate Smooth Monostratified Retinal Ganglion Cells
Source: eNeuro. 2024 Jan 25;11(1):ENEURO.0280-23.2023. doi: 10.1523/ENEURO.0280-23.2023 (PMC11078106; doi:10.1523/ENEURO.0280-23.2023)
Supplement: Extended Data Table 5-2. — Total bipolar cell synapses by type for smooth monostratified RGC (1321) and three parasol RGCs (5063, 5370, 18269) by type. Download Table 5-2, DOCX file. [file eneuro-11-ENEURO.0280-23.2023-s008.docx]

Extended Data Table 5-2. Total bipolar cell synapses onto parasol and smooth RGCs by type

| Neuron | DB4 | DB5 | DB6 | Giant | IMB | Unknown | Total |
| --- | --- | --- | --- | --- | --- | --- | --- |
| Smooth 1321 | 33 (26.2%) | 66 (52.4%) | 0 (0%) | 18 (14.3%) | 0 (0.0%) | 9 (7.1%) | 126 |
| Parasol 5063 | 81 (45.3%) | 29 (16.2%) | 0 (0%) | 1 (0.6%) | 65 (36.3%) | 3 (1.7%) | 179 |
| Parasol 5370 | 72 (47.7%) | 33 (21.9%) | 0 (0%) | 11 (7.3%) | 33 (21.9%) | 2 (1.3%) | 151 |
| Parasol 18269 | 122 (53.5%) | 41 (18.0%) | 1 (0.4%) | 13 (5.7%) | 45 (19.7%) | 6 (2.6%) | 228 |
