# Supplementary material for: Synaptic Origins of the Complex Receptive Field Structure in Primate Smooth Monostratified Retinal Ganglion Cells
Source: eNeuro. 2024 Jan 25;11(1):ENEURO.0280-23.2023. doi: 10.1523/ENEURO.0280-23.2023 (PMC11078106; doi:10.1523/ENEURO.0280-23.2023)
Supplement: Extended Data Table 7-3. — Number of ribbon synapses from each bipolar cell onto parasol RGC 18269. Download Table 7-3, DOCX file. [file eneuro-11-ENEURO.0280-23.2023-s011.docx]

Extended Data Table 7-3. Number of ribbon synapses from each bipolar cell onto parasol RGC 18269.

| Number of synapses | 1-2 | 3-4 | 5-7 | 8-10 | 11-14 | 15-33 | Total |
| --- | --- | --- | --- | --- | --- | --- | --- |
| DB4 | 4 | 1 | 0 | 0 | 3 | 3 |  |
| DB5 | 3 | 1 | 2 | 2 | 0 | 0 |  |
| DB6 | 1 | 0 | 0 | 0 | 0 | 0 |  |
| IMB | 8 | 6 | 2 | 0 | 0 | 0 |  |
| giant | 1 | 1 | 0 | 1 | 0 | 0 |  |
| TOTAL | 17 | 9 | 4 | 3 | 3 | 3 | 39 |
