# Supplementary material for: Synaptic Origins of the Complex Receptive Field Structure in Primate Smooth Monostratified Retinal Ganglion Cells
Source: eNeuro. 2024 Jan 25;11(1):ENEURO.0280-23.2023. doi: 10.1523/ENEURO.0280-23.2023 (PMC11078106; doi:10.1523/ENEURO.0280-23.2023)
Supplement: Extended Data Table 8-1. — Mixture of Gaussians model fit for the four clusters within the smooth monostratified RGC's ribbon synapses. The centroid values are the X and Y coordinates within the EM volume in microns where 0, 0 is the bottom left corner. Download Table 8-1, DOCX file. [file eneuro-11-ENEURO.0280-23.2023-s013.docx]

Extended Data Table 8-1. Mixture of Gaussians Model fit parameters.

| Component | Weight | Centroid | SD | Synapses | DB4 | DB5 | Giant |
| --- | --- | --- | --- | --- | --- | --- | --- |
| 1 | 0.238 | 208.37, 84.50 | 16.37, 19.26 | 21 | 7 | 10 | 4 |
| 2 | 0.314 | 132.04, 65.84 | 19.63, 17.42 | 30 | 7 | 23 | 0 |
| 3 | 0.150 | 94.10, 25.91 | 14.61, 6.10 | 14 | 4 | 8 | 2 |
| 4 | 0.297 | 125.47, 134.58 | 20.56, 17.20 | 18 | 5 | 15 | 8 |
